# Improvement of composite kidney outcomes by AKI care bundles: a systematic review and meta-analysis

**DOI:** 10.1186/s13054-023-04641-0

**Published:** 2023-10-09

**Authors:** Chun Yin See, Heng-Chih Pan, Jui-Yi Chen, Chun-Yi Wu, Hung-Wei Liao, Yen-Ta Huang, Jung-Hua Liu, Vin-Cent Wu, Marlies Ostermann

**Affiliations:** 1 Division of Nephrology, Department of Internal Medicine, National Cheng Kung University Hospital, College of Medicine, National Cheng Kung University, Tainan, Taiwan; 2 Graduate Institute of Clinical Medicine, College of Medicine, National Taiwan University, Taipei, 100 Taiwan; 3 Division of Nephrology, Department of Internal Medicine, Keelung Chang Gung Memorial Hospital, Keelung, 204 Taiwan; 4 College of Medicine, Chang Gung University, Taoyüan, 333 Taiwan; 5 Community Medicine Research Center, Keelung Chang Gung Memorial Hospital, Keelung, 204 Taiwan; 6 Division of Nephrology, Department of Internal Medicine, Chi-Mei Medical Center, Tainan, Taiwan; 7 Department of Health and Nutrition, Chia Nan University of Pharmacy and Science, Tainan, Taiwan; 8 Division of Nephrology, Department of Internal Medicine, Taichung Veterans General Hospital, Taichung, Taiwan; 9 Department of Nursing, Asia University, Taichung, Taiwan; 10 Division of Nephrology, Department of Internal Medicine, Wan Fang Hospital, Taipei Medical University, Taipei, 11696 Taiwan; 11 Division of Nephrology, Department of Internal Medicine, School of Medicine, College of Medicine, Taipei Medical University, Taipei, 11031 Taiwan; 12 Department of Surgery, College of Medicine, National Cheng Kung University Hospital, National Cheng Kung University, Tainan, Taiwan; 13 Department of Communication, National Chung Cheng University, Chiayi, Taiwan; 14 Department of Internal Medicine, Clinical Research Building, National Taiwan University Hospital, 7 Chung-Shan South Road, Room 1555, Taipei, Taiwan; 15 NSARF (National Taiwan University Hospital Study Group on Acute Renal Failure), Taipei, Taiwan; 16 Department of Critical Care, King’s College London, Guy’s and St. Thomas’ Foundation Trust, Westminster Bridge, London, SE1 7EH UK

**Keywords:** Acute kidney injury, Care bundles, Biomarkers

## Abstract

**Introduction:**

Various approaches have been suggested to identify acute kidney injury (AKI) early and to initiate kidney-protective measures in patients at risk or with AKI. The objective of this study was to evaluate whether care bundles improve kidney outcomes in these patients.

**Methods:**

We conducted a systematic review of the literature to evaluate the clinical effectiveness of AKI care bundles with or without urinary biomarkers in the recognition and management of AKI. The main outcomes were major adverse kidney events (MAKEs) consisting of moderate-severe AKI, receipt of renal replacement therapy (RRT), and mortality.

**Results:**

Out of 7434 abstracts screened, 946 published studies were identified. Thirteen studies [five randomized controlled trials (RCTs) and eight non-RCTs] including 16,540 patients were eligible for inclusion in the meta-analysis. Meta-analysis showed a lower incidence of MAKE in the AKI care bundle group [odds ratio (OR) 0.73, 95% confidence interval (CI) 0.66–0.81] with differences in all 3 individual outcomes [moderate–severe AKI (OR 0.65, 95% CI 0.51–0.82), RRT (OR 0.63, 95% CI = 0.46–0.88) and mortality]. Subgroup analysis of the RCTs, all adopted biomarker-based approach, decreased the risk of MAKE (OR 0.55, 95% CI 0.41–0.74). Network meta-analysis could reveal that the incorporation of biomarkers in care bundles carried a significantly lower risk of MAKE when compared to care bundles without biomarkers (OR = 0.693, 95% CI = 0.50–0.96), while the usual care subgroup had a significantly higher risk (OR = 1.29, 95% CI = 1.09–1.52).

**Conclusion:**

Our meta-analysis demonstrated that care bundles decreased the risk of MAKE, moderate–severe AKI and need for RRT in AKI patients. Moreover, the inclusion of biomarkers in care bundles had a greater impact than care bundles without biomarkers.

**Supplementary Information:**

The online version contains supplementary material available at 10.1186/s13054-023-04641-0.

## Take-home message

This systematic review highlighted the impact of care bundles in reducing the risk of major adverse kidney events (MAKE) in patients at risk or with acute kidney injury (AKI). An additional benefit of biomarker inclusion for early AKI recognition and management was demonstrated by network meta-analysis.

## Introduction

Acute kidney injury (AKI) is a common complication among hospitalized patients, especially those with critical illness or undergoing major surgery. However, the reported occurrence of AKI varies widely due to differences in patients’ baseline characteristics, type of surgery, acute and chronic comorbidities and AKI definition and diagnostic criteria. AKI occurrence in the intensive care unit (ICU) commonly exceeds 50%, with septic shock being the most frequent reported etiology. The association with mortality (including ICU mortality, hospital mortality, 28-day and 90-day mortality) was also highly variable, ranging from 11 to 77% [1–3]. The incidence of AKI in cardiac surgery patients broadly ranges from 3.1 to 39.9% [4]. A retrospective cohort study involving 4229 patients undergoing major non-cardiac surgery showed that the incidence rose from 8.1 to 64.0% if both serum creatinine and urine output were included in the AKI diagnosis compared to using serum creatinine alone [5]. While the majority of patients will recover renal function, there is increasing evidence that AKI has serious short- and long-term complications, including an increased risk of dialysis-dependent chronic kidney disease (CKD), major adverse cardiovascular events (MACEs) and mortality [6, 7]. The risk is higher in patients with more severe and more prolonged AKI and in those with preexisting CKD. Further, patients with subclinical AKI (defined by elevation of kidney biomarkers without meeting serum creatinine or urine output criteria for AKI) and patients with initial recovery of renal function after AKI also remain at risk of kidney disease progression [8]. Early recognition of AKI and appropriate and timely management, including avoidance of further nephrotoxic insults, are the mainstay strategies to prevent progression to CKD and unfavorable outcomes.

The 2012 Kidney Disease Improving Global Outcomes (KDIGO) AKI guideline includes recommendations to prevent and manage AKI, including optimization of hemodynamics and fluid status, prevention of nephrotoxic insults and avoidance of hyperglycemia. Collectively, they are often referred to as “AKI care bundle” although the essential components and specific targets have not been standardized.

AKI is defined by the KDIGO criteria based on serum creatinine and urine output. However, both are relatively late markers and not specific for AKI. Numerous new biomarkers that indicate AKI earlier than serum creatinine are available, such as cell cycle arrest markers like tissue inhibitor of metalloproteinases 2 (TIMP-2) and insulin-like growth factor-binding protein 7 (IGFBP7), and neutrophil gelatinase-associated lipocalin (NGAL). TIMP-2 and IGPBP7 are released during tubular cell cycle arrest and detectable in the urine within 1–2 h of tubular stress. NGAL is a marker for kidney damage, which can be detected as early as 3 h in the urine after ischemic or nephrotoxic kidney injury [9, 10]. TIMP-2 and IGFBP7 have been used for enrichment purposes in studies exploring the role of AKI care bundles. Kapoor et al. [11] measured NGAL before and after goal-directed optimization but did not utilize the results to identify high-risk patients or guide management.

The purpose of this study is to compare the effectiveness of AKI care bundles with and without biomarker-guided stratification to usual care through a systematic review and meta-analysis.

## Materials and methods

### Data sources and search strategy

This systematic review and meta-analysis were conducted according to the Preferred Reporting Items for Systematic Reviews and Meta-Analyses (PRISMA) guidelines (Additional file 1: Table S1 and S2). The protocol was registered within the International Platform of Registered Systemic Review and Meta-analysis Protocols (INPLASY202370043). We performed a comprehensive literature search using PubMed, EMBASE, and Cochrane Library to identify all studies published since 2012 that had included AKI care bundles. The following key terms were included: “acute kidney injury”, “acute kidney failure”, “acute renal failure”, “urinary biomarker”, “neutrophil gelatinase associated lipocalin”, “NGAL”, “tissue inhibitor of metalloproteinase 2”, “tissue inhibitor of matrix metalloproteinase 2”, “TIMP-2·IGFBP7”, “care bundle”, “renal replacement therapy”, “mortality”, “randomized controlled trial”, “cohort analysis”, and “cohort study”.

### Inclusion and exclusion criteria

This study assessed the clinical use of AKI care bundles with or without biomarkers, participants aged 18 years or older of any ethnic origin or sex, and published in English. The exclusion criteria were as follows: (1) studies including patients with preexisting advanced CKD [estimated glomerular filtration rate (eGFR) ≤ 30 mL/min/1.73 m^2^], chronic dialysis-dependence or previously received dialysis, or kidney transplantation; (2) studies including pregnant or lactating patients; and (3) editorials, letters, review articles, conference or case reports. Only fully published papers were selected for quality assessment and data synthesis.

### Study selection and data extraction

The search results were independently reviewed by two investigators (CYS and HWL), and eligible studies were selected. A third investigator (VCW) helped resolving any disagreement that arose. Relevant data were independently extracted from the included studies by the first investigator (CYS) using an agreed standardized template. The data included information about the studies (author name, year of publication, setting, population, care bundle type, sample size, study endpoints) and details about the study participants [average age (years), gender (%), comorbidities]. The odds ratios (ORs) and 95% confidence intervals (CI) were extracted when available. Other data that were predetermined included the intensive care unit (ICU) type (surgical/mixed or medical), diagnostic criteria for AKI and moderate–severe AKI, cohort size, and presence of sepsis. Any potential differences in data extraction were handled by two investigators (CYS and VCW).

### Outcomes

The aims of this analysis were to (a) investigate the effectiveness of AKI care bundles and (b) to compare biomarker-guided care bundles with bundles that did not incorporate new AKI biomarkers. The main patient outcomes were MAKE including moderate–severe AKI (defined as KDIGO AKI stage 2 to 3), receipt of renal replacement therapy (RRT), or mortality.

### Pre-specified subgroup analysis

We hypothesized that the incorporation of AKI biomarkers in care bundles impacted patient outcomes.

### Statistical analysis

We used the Review Manager software package (RevMan) version 5.4.1 (The Nordic Cochrane Centre, Copenhagen, Denmark, 2020) for outcome analyses. Forest plots of the outcomes were created using the Mantel–Haenszel statistical method and random effect analysis model due to the diverse methodologies used in the included studies. Funnel plots were constructed to examine any exaggeration of effect estimates from low-quality studies. Risk of bias was assessed by RoB 2.0 (a revised tool to assess risk of bias in randomized trials) according to the Cochrane Handbook for Systematic Reviews of Interventions Version 6.3, 2022 [12] and Newcastle–Ottawa Scale (NOS) for Assessing the Quality of Non-Randomized Studies [13]. Heterogeneity was quantified by *I*^2^ statistic. The extent of heterogeneity was categorized into mild (*I*^2^ < 30%), moderate (30 ≤ *I*^2^ < 50%), and substantial (*I*^2^ > 50%). Network meta-analysis (NMA) was employed for pairwise comparison between patients with and without care bundles and stratification based on biomarkers. We used MetaInsight V4.0.0 [National Institute for Health and Care Research (NIHR)—Complex Reviews Support Unit (CRSU), United Kingdom, 2023] [14], a tool adapted from the R software to conduct NMA. Surface under the cumulative ranking curve (SUCRA) was used to show the hierarchy of the treatment effects in a rank-heat plot, with the preferential treatment having the highest SUCRA value. Trial sequential analysis (TSA) was employed to reduce the likelihood of type 1 and 2 errors after repetitive significance analysis of the study data (Copenhagen Trial Unit, Centre for Clinical Intervention Research, Denmark, 0.9.5.10 Beta software). This statistical methodology also assesses the need for further trials to clarify the effect of an intervention [15, 16]. TSA was used to confirm the impact of biomarker incorporation in AKI care bundles.

## Results

### Study selection and data characteristics

**Fig. 1:**
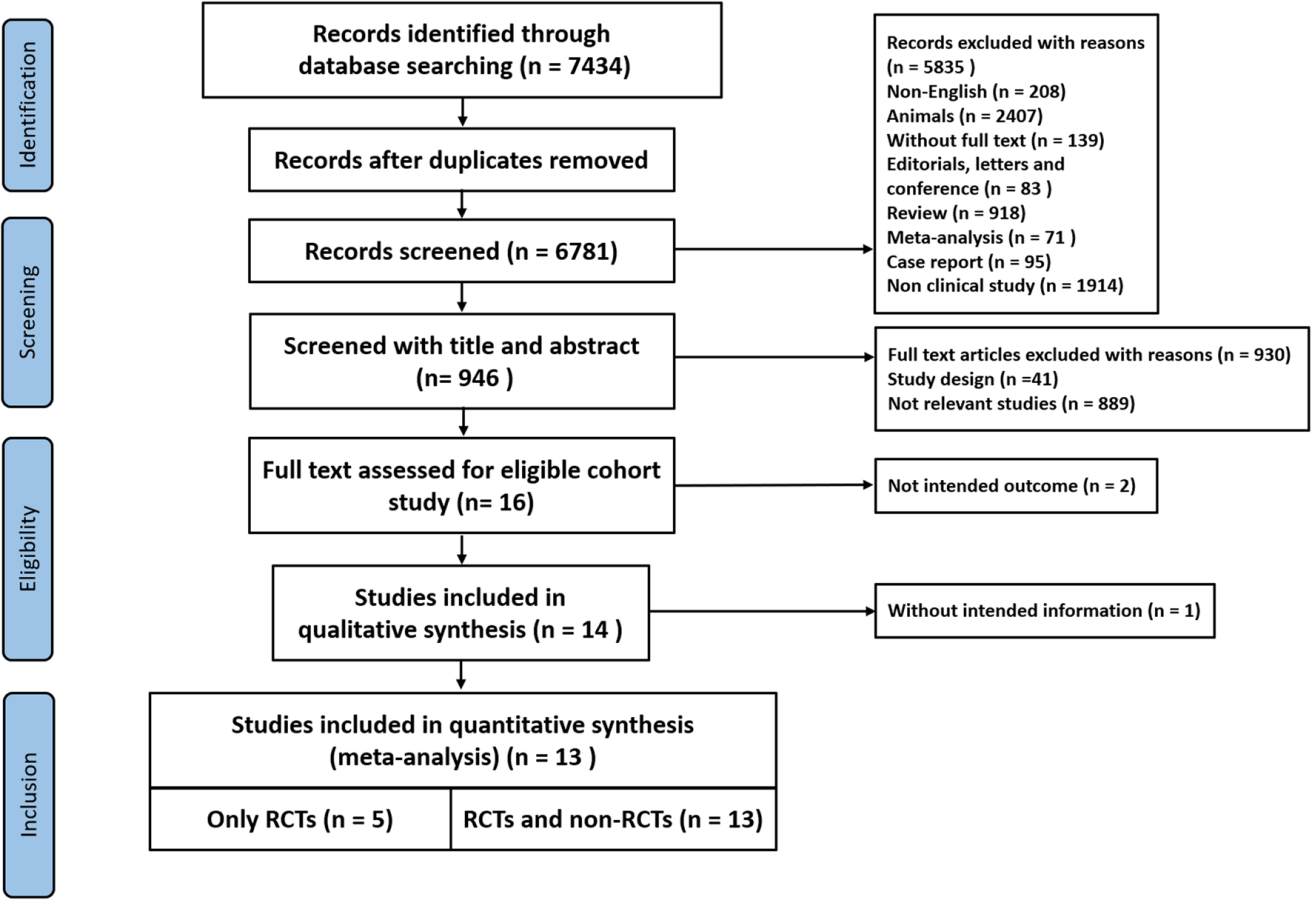
PRISMA flow diagram of study selection. *PRISMA* Preferred Reporting Items for Systematic Reviews and Meta-Analyses

The initial search revealed 7434 studies of which thirteen were included for further analysis (including a total 6433 patients). We excluded studies that were duplicates or met other exclusion criteria (Fig. 1**)**.

Five of the selected articles were randomized controlled trials (RCTs) [11, 17–20], and the remaining were prospective interventional studies (*n* = 1) [21], prospective observational studies (*n* = 3) [22–24], retrospective before-and-after cohort studies (*n* = 3) [25–27] and a propensity score-matched cohort study [28]. Of the thirteen included studies, nine studies were performed in surgical patients of which five involved only patients undergoing cardiac surgery. Eight studies (including all five RCTs) incorporated urinary biomarkers [11, 17–20, 25–27] of which seven utilized [TIMP-2] and [IGFBP7] to identify patients at higher risk of AKI after major surgery or during critical illness [17–20, 25–27]. In studies that used [TIMP-2]·[IGFBP7] (Nephrocheck^®^ Test, Astute Medical, San Diego, California, USA), patients were classified as high risk if the urinary [TIMP-2]·[IGFBP7] value exceeded 0.3 (ng/mL)^2^/1000 [29]. Only Kapoor et al. [11] (a RCT) measured NGAL and demonstrated differences in urinary NGAL before and after coronary artery surgery, and in patients receiving conventional or goal-directed therapy. The timing of biomarker measurement differed among the studies. In most studies, the biomarkers were measured at four hours after surgery [18, 20, 25] or immediately after ICU admission [11, 17, 19, 27]. Kapoor et al. measured serum and urinary NGAL at different timepoints (baseline, postoperative, 4 h and 24 h postoperatively) and found that the elevation of urinary NGAL at 4 h after cardiac surgery correlated highly with serum creatinine and AKI [11]. The studied populations were limited to patients in the critical care setting or patients undergoing major surgery. The relevant care bundles applied to the intervention cohorts were either based on the KDIGO AKI recommendations or included goal-directed management algorithms representing modified versions of the KDIGO guideline (Additional file 1: Table S3). The individual components of the care bundles were adopted or modified from the KDIGO recommendations, such as avoidance of nephrotoxins and radiocontrast agents, hemodynamic monitoring and optimization of fluid status (Additional file 1: Table S3). The study endpoints were highly variable between the studies, comprising of occurrence of different stages of AKI, receipt of RRT, length of ICU and hospital stay, ICU or hospital mortality and survival at different time points (e.g., 30, 60, or 90 days) (Table 1).

**Table 1:** Characteristics of included comparative studies for outcome evaluation

No.	References	Study design	Population setting	Total patient	Type of care bundles	Use of biomarker and timing of measurement	Primary study endpoints
1	Kolhe et al. [24]	Prospective observational study	All adult patients who were admitted with or developed AKI, in any location of the hospital	2297	KDIGO care bundle	NA	Proportion of AKI episodes with progression to higher AKI stage, length of stay, in-hospital case fatality, 30-day case fatality, 60-day case fatality
2	Kolhe et al. [28]	Propensity score-matched cohort study	All adult patients who were admitted with or developed AKI, in any location of the hospital	3717	AKI bundle derived from National Confidential Enquiry into Patient Outcome and Death (NCEPOD)	NA	All-cause in-hospital case fatality
3	Meersch et al. [18]	Randomized controlled trial	Patients who underwent cardiac surgery with the use of CPB	276	KDIGO cardiothoracic surgery bundle	Urinary [TIMP-2]·[IGFBP7] at 4 h after CPB	AKI within the first 72 h after surgery
4	Göcze et al. [17]	Randomized controlled trial	Patients who underwent major non-cardiac surgery	121	KDIGO care bundle	Urinary [TIMP-2]·[IGFBP7] at intensive care unit admission	AKI during the first 7 days after surgery
5	Kapoor et al. [11]	Randomized controlled trial	Patient underwent on-pump coronary artery surgery	110	Goal-directed therapy	Plasma and urinary NGAL, before initiation of surgery, on arrival in the ICU, and 4 h and 24 h later	Plasma and urinary NGAL postoperatively and at 4 h and 24 h after surgery
6	Schanz et al. [19]	Randomized controlled trial	Critically ill patients admitted to the monitoring ward, or fulfilling systemic inflammatory response syndrome (SIRS) criteria, or patient triaged for immediate or highly urgent treatment based on the Manchester Triage	100	KDIGO care bundle	Urinary [TIMP-2]·[IGFBP7] on admission	Incidence of moderate-severe AKI within the first day after admission
7	Engelman et al. [26]	Before-and-after study	Patient who underwent on-pump cardiac surgery	847	KDIGO care bundle	Urinary TIMP-2·IGFBP7, at the morning after cardiac surgery	Development of stage 2 or 3 AKI
8	Koeze et al. [23]	Before-and-after study	Patients in the ICU	2642	“Save the kidney” educational intervention bundle adapted from KDIGO	NA	Composite of mortality, RRT, and progression of AKI
9	Zarbock et al. [20]	Randomized controlled trial	Patients who underwent cardiac surgery	278	KDIGO care bundle	Urinary [TIMP-2]·[IGFBP7] at 4 h after CPB	Compliance rate to KDIGO bundle
10	Halmy et al. [27]	Before-and-after study	Patients who underwent major non-cardiac surgery	294	KDIGO care bundle	Urinary TIMP-2·IGFBP7, on ICU admission	Early AKI recovery, i.e., complete reversal of any AKI stage to absence of AKI within the first 7 postoperative days
11	Couturier et al. [25]	Before-and-after study	Patients who underwent elective cardiac surgery	461	Renal supportive measures according to an ICU local protocol based on KDIGO guidelines	Urinary TIMP-2·IGFBP7, at 4 h after the end of the procedure in the interventional cohort	All AKI within 48 h
12	Bourdeaux et al. [22]	Prospective observational study	Patients in the ICU	4783	KDIGO care bundle	NA	The proportion AKI patients developing a worse stage of AKI during their stay
13	Kotwal et al. [21]	Prospective interventional study	The majority of patients were located in general hospital wards (94.7%)	614	“STOP AKI” management guideline	NA	Length of stay and all-cause in-hospital mortality

*AKI* Acute kidney injury; *CPB* Cardiopulmonary bypass; *ICU* Intensive care unit; *KDIGO* Kidney Disease Improving Global Outcomes; *NA* Not applicable; *RRT* Renal replacement therapy

### Quality of included studies

The publication years, sample sizes (100–5044 patients) and characteristics of the study population of the 13 studies differed (Table 1–2). A critical appraisal demonstrated a relatively high performance and detection bias. Additional file 1: Fig. S1 shows a comprehensive risk of bias graph. The RoB 2 and NOS revealed that the risk of bias of the included studies varied. In each study, there was a low and/or unclear risk in most domains of bias evaluation. The risk of bias was low for random sequence generation in 9 studies (69.2%); allocation concealment in 8 studies (61.5%); blinding of outcome assessment in only 1 study (7.7%); incomplete outcome data in 8 studies (61.5%); and selective reporting in 10 studies (76.9%). No study had any other bias. Therefore, according to the criteria of overall quality, 8 studies (61.5%) were rated as low risk, 1 study (7.7%) as unclear risk, and 4 studies (30.8%) as high risk. TSA of composite kidney outcomes was conducted for assessment of the statistical reliability of included data, given the limitations of relatively restricted sample sizes. We calculated the required information size (RIS) according to assumptions and goals. A 2500 event rate was assumed in the control arm, which was roughly the median of included studies. A 28.6% reduction in relative risk, equivalent to a 5% reduction in absolute risk, was considered a clinically meaningful effect of the intervention (care bundles). A type 1 error of 5% and a power of 90% were adopted. The heterogeneity adjusted RIS was calculated to be 2629 patients. The cumulative Z-curve surpassed the conventional boundary for statistical significance and the trial sequential monitoring boundary for benefits. Moreover, the accumulated case number of included studies was larger than RIS, indicating that the current evidence reached a conclusion supporting the superior performance of care bundles over usual treatment in composite renal outcomes (Additional file 1: Fig. S2).

**Table 2:** Characteristics of study population in the included comparative studies

No.	References	Age (mean)	Male (%)	Ethnicity (Caucasian%)	Type of surgery	Diabetes (%)	eGFR***	Risk score
1	Kolhe et al. [24]	76.9	44.4	91.4	NA	Unknown	NA	Charlson’s score (score ≤ 2 in 68% patients)
2	Kolhe et al. [28]	76.4	48.9	90.3	Included both surgical and medical patients	Unknown	NA	NA
3	Meersch et al. [18]	68.4	68.1	Unknown	Cardiac surgery	25.4	NA	SOFA score: (control) 6.0 ± 2.2; (intervention) 5.9 ± 2.1 APACHE score: (control) 8.9 ± 3.9; (intervention) 8.5 ± 3.2 EuroSCORE: (control) 5; (intervention) 6
4	Göcze et al. [17]	63	73.3	Unknown	Major elective non-cardiac surgery	Unknown	> 15	SAPS II, median (IQR): (standard care) 32 (24.5–38); (intervention) 31 (22–38)
5	Kapoor et al. [11]	55.5	39	Unknown*	On-pump coronary artery bypass graft surgery	13	> 15	EuroSCORE: (control) 3.21 ± 0.97; (GDT) 3.11 ± 0.79
6	Schanz et al. [19]	66.4	44.4	Unknown	NA	18.5	Not undergoing renal replacement therapy	SOFA score, median (IQR): (control) 5 (3–10); (intervention) 7.5 (4.5–8.3) APACHE II score, median (IQR): (control) 15 (12–20.5); (intervention) 18 (10.25–24.5) SAPS II, median (IQR): (control) 39 (29–45); (intervention) 36.5 (26.5–61.0)
7	Engelman et al. [26]	66.3	75.5	93.4**	Cardiac surgery	38.9	Creatinine < 2.0 mg/dL	NA
8	Koeze et al. [23]	59.6	62	Unknown	Included both surgical and medical patients	15	Creatinine < 2.0 mg/dL	APACHE IV score, mean ± SD: (control) 51 (25); (intervention) 52.3 (25)
9	Zarbock et al. [20]	66.9	69.1	Unknown	Cardiac surgery with cardiopulmonary bypass	25.7	> 20	SOFA score, mean ± SD: (control) 9.6 (3.4); (intervention) 10.2 (3.1) APACHE score, median (IQR): (control) 20 (12.5, 22); (intervention) 20 (17.5, 23)
10	Halmy et al. [27]	64	59	Unknown	Major surgery	20	> 15	SAPS II, median (IQR): (historical control) 30 (23–38.5); (protocol implementation) 30 (24.25–37.75)
11	Couturier et al. [25]	66	71	Unknown	Cardiac surgery	26	> 30	SOFA score: (control) 6.9 ± 2.5; (TIMP2*IGFBP7) 5.9 ± 2.1 EuroSCORE: (control) 2.4 ± 3.2; (TIMP-2·IGFBP7) 2.3 ± 2.9
12	Bourdeaux et al. [22]	63	32.2	98.7	Included both surgical and medical patients	Unknown	Not undergoing renal replacement therapy	APACHE II/EuroScore: General ICU (control) 15.3 ± 7.1; (intervention) 15 ± 6.6 Cardiac ICU (control) 5.3 ± 2.9; (intervention) 8.9 ± 8.2
13	Kotwal et al. [21]	74	56	Unknown	NA	31	Not undergoing renal replacement therapy	NA

*APACHE* Acute Physiology And Chronic Health Evaluation; *CKD* Chronic kidney disease; *GDT* Goal-directed therapy; *ICU* Intensive care unit; *IQR* Interquartile range; *NA* Not applicable; *SAPS* Simplified Acute Physiology Score; *SD* Standard deviation; *SOFA* Sequential Organ Failure Assessment

*The study was conducted in a tertiary care hospital located at New Delhi, Delhi, India

**Total number of Caucasian patients in the urinary biomarker/acute kidney response team

***Patients with more advanced CKD were excluded from the studies

### Incidence of major adverse kidney events (MAKE)

**Fig. 2:**
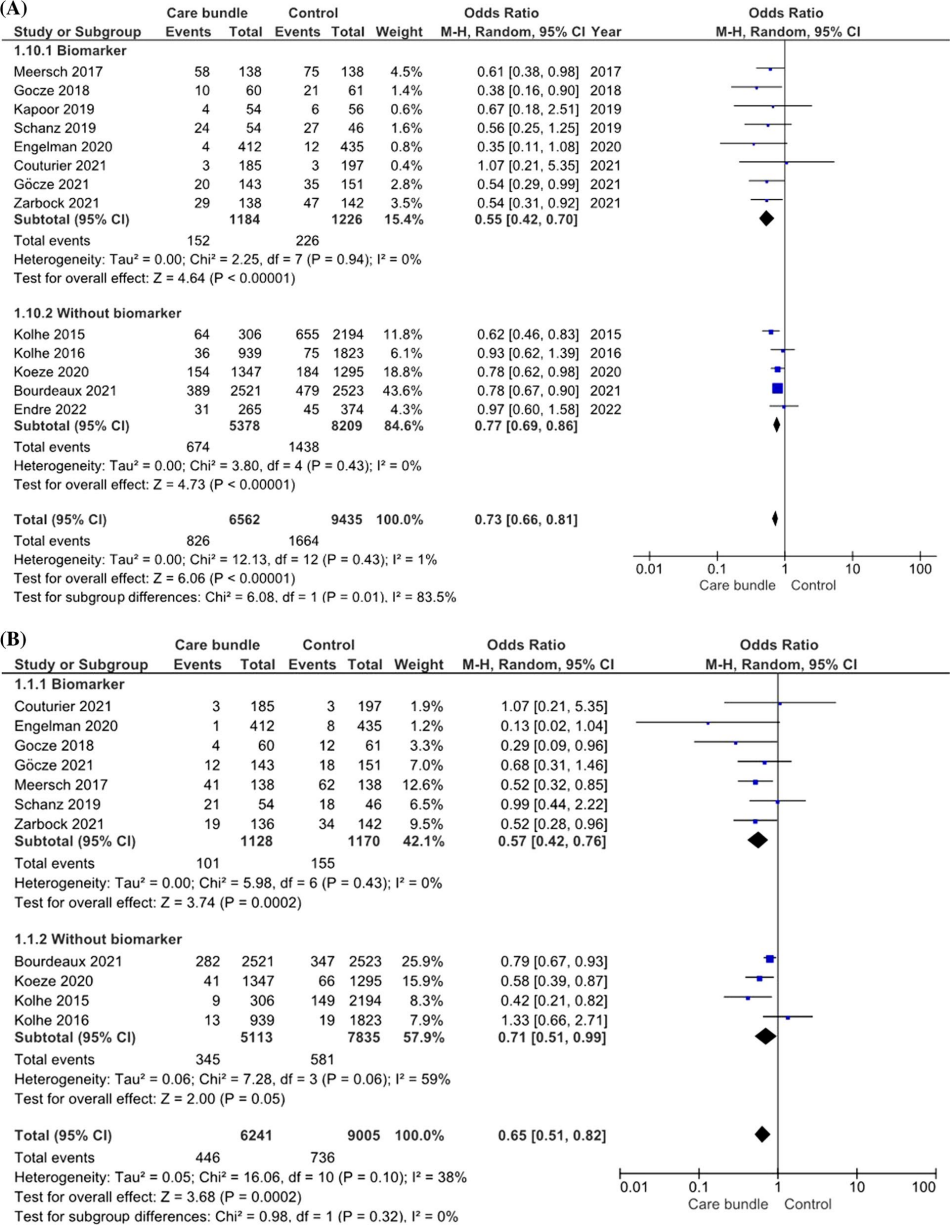

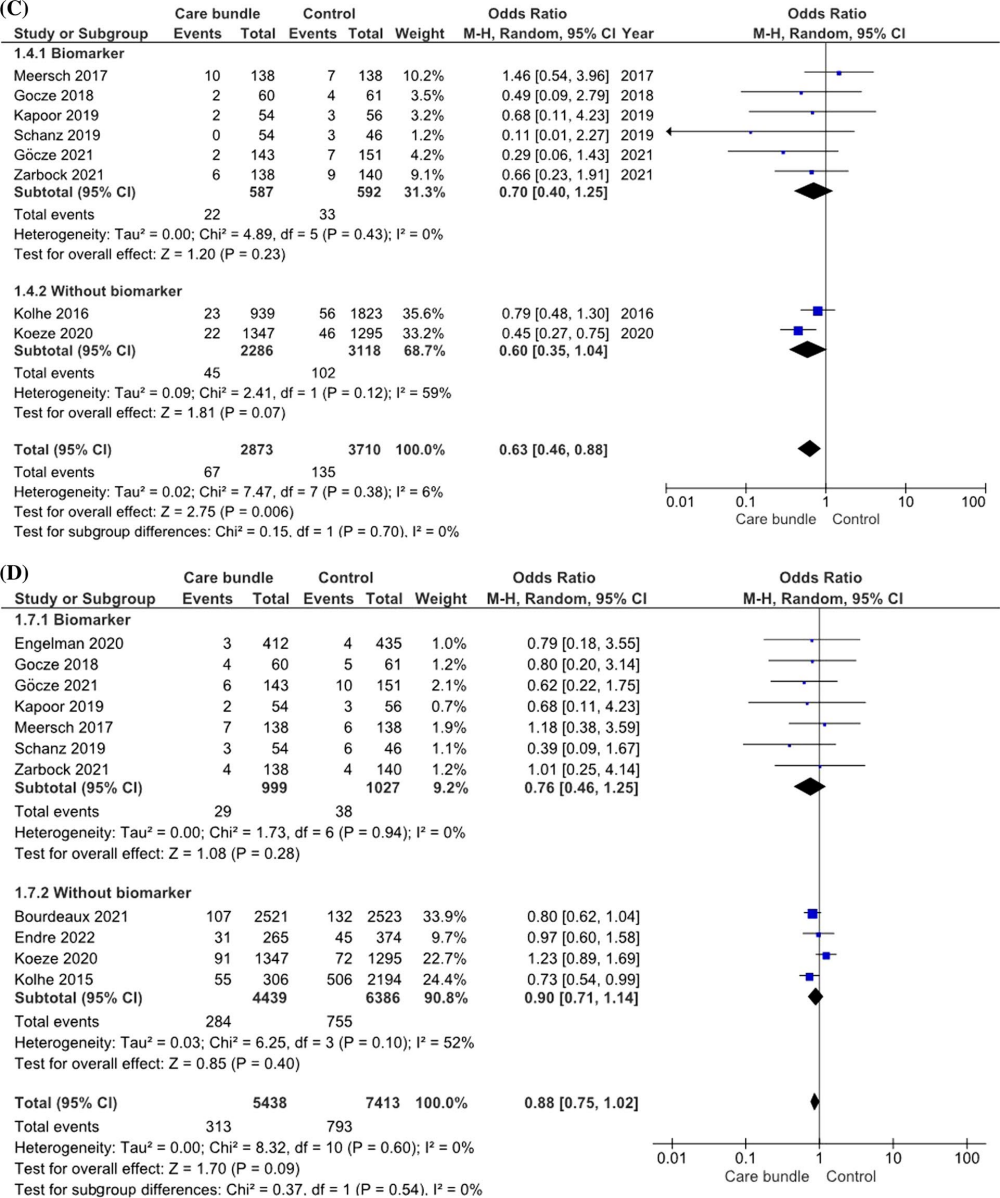
Forest plot stratified the risk of **a** major adverse kidney events (MAKE), **b** acute kidney injury (AKI), **c** renal replacement therapy (RRT), and **d** all-cause mortality associated with care bundle versus usual care. All randomized controlled trials (RCTs) and non-RCTs that met the inclusion criteria were included in the analysis. **a** Forest plot of MAKE from care bundle versus usual care separated based on whether they included biomarkers. **b** Forest plot of AKI from care bundle versus usual care separated based on whether they included biomarkers. **c** Forest plot of RRT from care bundle versus usual care separated based on whether they included biomarkers. **d** Forest plot of all-cause mortality from care bundle versus usual care separated based on whether they included biomarkers

**Fig. 3:**
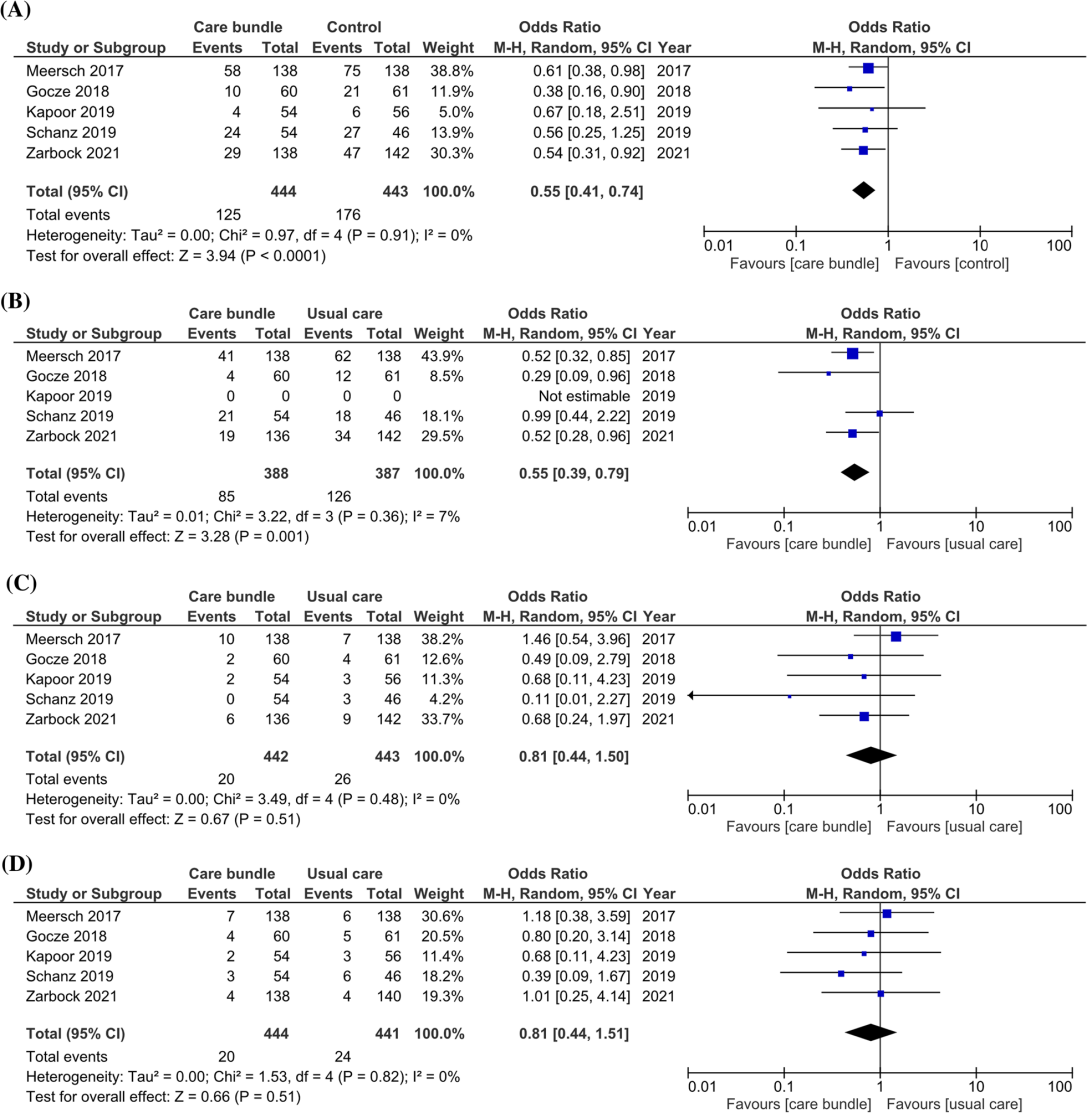
Forest plot stratified the risk of **a** major adverse kidney events (MAKEs), **b** acute kidney injury (AKI), **c** renal replacement therapy (RRT), and **d** all-cause mortality associated with care bundle versus usual care. Only randomized controlled trials (RCTs) that met the inclusion criteria were included in the analysis. **a** Forest plot of MAKEs from care bundle versus usual care with all RCTs. **b** Forest plot of AKI from care bundle versus usual care with all RCTs. **c** Forest plot of RRT from care bundle versus usual care with all RCTs. **d** Forest plot of all-cause mortality from care bundle versus usual care with all RCTs

The primary outcome of interest was MAKE based on all included studies including a total of 16,540 patients with 17,004 AKI events. The pooled incidence of MAKE was 12.6% (826/6562) in the groups of patients in whom care bundles were applied versus 17.6% (1664/9435) in patients receiving usual care. Being managed according to a care bundle was associated with a significant survival benefit compared to receiving usual care (OR of 0.73; 95% CI 0.66–0.81; *p* < 0.001) (Fig. 2A). There was a low degree of heterogeneity among the studies (random effect model, I2 value of 1%). Additionally, subgroup analysis of RCTs only showed that the OR for MAKE was 0.55 (95% CI 0.41–0.74; *p* < 0.001) (Fig. 3A). We further assessed the impact of utilizing AKI biomarker on the association between AKI care bundles and the reduced risk of MAKE. Analyzing biomarker-guided and non-biomarker-guided studies separately, the benefit of care bundles over usual care was seen in both groups. However, using additional kidney biomarkers to identify AKI earlier and initiate care bundles lowered the MAKE risk by 45% compared to usual care; care bundles without incorporation of kidney biomarkers reduced the risk by 23%.

### Progression of AKI and need of RRT

All 13 included trials provided detailed information on the occurrence of moderate–severe AKI and receipt of RRT. The pooled incidence of moderate–severe AKI was 7.1% in the intervention cohort versus 8.2% in the usual care group. There was an overt reduction in the pooled risk of moderate–severe AKI between the care bundle and usual care groups with an OR of 0.65 (95% CI 0.51–0.82; *p* < 0.001) using the random effect model (*I*^2^ value = 38%) (Fig. 2B). Compared to usual care, the application of care bundles that incorporated AKI biomarkers lowered the risk of progression to moderate–severe AKI by 43% compared to 35% reduction when using care bundles without AKI biomarkers. Similar trend was noticed in the subgroup analyses focusing on patients who underwent cardiovascular surgery (Additional file 1: Fig. S4).

Eight trials provided detailed information related to initiation of RRT. Figure 2C shows a significant difference in risk of RRT between the care bundle and usual care group (OR = 0.63; 95% CI 0.46–0.88, *p* = 0.006) using a random effect model with low heterogeneity (*I*^2^ value = 6%). However, the association between utilization of care bundles and reduction of RRT risk was no longer significant when comparing the biomarker- and non-biomarker-guided care bundle groups separately with usual care [OR 0.70; 95% CI 0.40–1.25 (*p* = 0.43) and OR 0.60; 95% CI 0.35–1.04 (*p* = 0.12), respectively].

### Mortality

Pooled data of 11 studies showed a mortality of 6% in the care bundle group versus 11.1% in the usual care group. The difference was statistically not significant [log OR 0.88; 95% CI = 0.75–1.02; *p* = 0.09 using a random effect model (*I*^2^ value = 0%)] (Fig. 2D). This finding remained consistent when analyzing studies with biomarker-guided versus non-biomarker-guided care bundles separately.

### Network meta-analyses (NMA)

To explore the impact of biomarker containing care bundles on risk of MAKE, NMA was conducted with all thirteen studies. Studies using biomarker-guided care bundles could report a significantly lower risk of MAKE compared to studies with care bundles that did not incorporate biomarker enrichment (OR 0.693; 95% CI 0.50–0.96; *p* < 0.05), while the usual care subgroup had a relatively higher risk (OR 1.29; 95% CI 1.09–1.52; *p* < 0.05) (Additional file 1: Fig. S5). SUCRA plots illustrated that biomarker-guided care bundles provided best outcomes, followed by non-biomarker-guided care bundles and usual care (Additional file 1: Fig. S6).

### Publication analysis

Publication bias was assessed visually using funnel plots. Additional file 1: Fig. S3 shows a symmetrical funnel plot with a narrow base, indicating minimal publication bias in this meta-analysis.

### Assessment of quality of evidence and summary of findings

The quality of evidence was assessed using the GRADE system. We evaluated the primary outcomes and presented them as a summary of findings in Additional file 1: Appendix.

## Discussion

This systematic review and meta-analysis concluded that the implementation of AKI care bundles was associated with a significantly reduced risk of MAKE. Strategies enriched by kidney biomarkers to enable early identification of AKI and to guide the initiation of AKI care bundles could be more effective than using care bundles that did not incorporate new biomarkers.

Our analysis showed that some of the care bundles used in the literature contained more than 5 elements (Additional file 1: Table S3). It is generally assumed that all components in a care bundle have equal weighting. However, *Groote* et al. showed that the components of the care bundle used in the PrevAKI trial (*Meersch* et al.) had differential impact on the risk of AKI post-cardiac surgery [18, 30]. For instance, reversal of hypotension and low cardiac output was more effective than avoidance of hyperglycemia or contrast exposure. Whether this also applies to patients undergoing major non-cardiac surgery is currently unknown.

The risk of MAKE was significantly lower in the intervention group. Our subgroup analyses exploring the role of biomarkers add to the previously published meta-analysis on AKI care bundles and recent consensus recommendations [31–33]. [TIMP-2] and [IGFBP7] are the only two urinary AKI biomarkers that are currently approved by the United States Food and Drug Administration (USFDA). Urinary [TIMP-2]·[IGFBP7] were the most commonly used biomarkers in the studies identified in our literature search [11, 17–20, 25–27]. Biomarker testing was done in study populations that were at high risk of AKI, such as hemodynamically unstable and critically ill patients and patients undergoing major surgery. In these cohorts, urinary cell cycle arrest markers indicate tubular cell stress and the magnitude of elevation correlates with the extent of tubular cell stress / damage and risk of AKI [34].

In most studies, the cutoff value to define high-risk patients was a urinary [TIMP-2]·[IGFBP7] value of 0.3 or greater [11, 17–20, 25, 26]. However, the intervention strategies differed between studies. For instance, Halmy et al. stratified patients into low-risk ([TIMP-2]·[IGFBP7] < 0.3), moderate-risk ([TIMP-2]·[IGFBP7] = 0.3–2.0) and high-risk ([TIMP-2]·[IGFBP7] > 2.0) subgroups and tailored the interventions accordingly. Optimization of fluid status and avoidance of nephrotoxic agents were included in the intervention protocols for all 3 risk groups, whereas more invasive hemodynamic monitoring, such as measurement of mixed venous oxygen saturation (SvO2), was only done in the high-risk AKI subgroup. Thirty percentage of patients (14/46) in the low-risk subgroup developed AKI with 7-day recovery rate of 78%, while 33% of patients (32/97) in the high-risk AKI subgroup had AKI of whom 79% recovered within 7 days [27]. Thus, inclusion of biomarkers into the treatment algorithm allows enrichment and provides opportunities for timely intervention to improve outcomes, as confirmed in this meta-analysis.

Intriguingly, our results showed that the incorporation of biomarkers in care bundles did not have a significant impact on the application of RRT and mortality. However, none of the studies were powered for these outcomes. Further, the application of AKI care bundles has potentially unintended effects, but the data are sparse. For instance, the implementation of AKI care bundles may increase the workload of the clinical team. Additionally, most of the KDIGO recommendations are based on expert opinion rather than robust evidence, with only 14.8% graded as “1A” [35]. This should be remembered when implementing AKI care bundles and a tailored approach may be considered in individual patients [36].

A sustained favorable outcome of AKI care bundles was demonstrated with the aid of TSA. Additionally, the monitoring boundary of benefit was surpassed consistently, indicating a consistent beneficial effect with the use of care bundles for patients at high risk of AKI.

The main limitation of this meta-analysis is the generalizability. We acknowledge that the results are based on studies that were conducted in the critical care and emergency setting, and more than half of the studies were performed post-cardiac surgery. Only one study was conducted in AKI patients outside the ICU [21]. Further, the majority of study participants were Caucasian, with only one study completed in Asia [11]. Second, the studies were heterogenous in design and explored different outcomes. For example, mortality rate was reported inconsistently and at different time points. It is possible that our method of pooling and re-analyzing may have missed a significant effect on mortality risk related to care bundles. Nevertheless, by using the *I*^2^ statistics, the heterogeneity among the included studies was negligible when they were separated based on whether they included biomarkers in the care bundle compared to usual care. The pooled results are meaningful and clearly showed a trend toward mortality reduction. However, more studies are needed to validate this outcome. Third, we were not able to perform a detailed analysis of the side effect profile of AKI care bundles due to the lack of data in the published studies. Nevertheless, it should be acknowledged that all results related to the roles of biomarker-guided AKI care bundle were derived from RCTs, while data related to care bundles without biomarkers stemmed mostly from non-RCTs, which may introduce selection bias.

Fourth, simple interpretation of the SUCRA ranking could cause exaggeration of the treatment effect if the comparison was performed without further validation such as calculation of the normalized entropy [37]. Our analysis merely compared three intervention strategies, and a distinctive cumulative probability graph was depicted. The implementation of biomarker-guided care bundles was associated with better outcomes than using care bundles without biomarker enrichment or usual care. The relationship between these 3 strategies was unequivocally certain (Additional file 1: Fig. S6B). Fifth, we acknowledge a high risk of bias. The randomization process used in the RCTs was described clearly in the methodology (e.g., by block randomization or sequential randomization), but not every study attempted to conceal the allocation process. *Gocze* et al. and Kapoor et al. allocated their study participants by a sealed envelope technique [11, 17], while *Meersch* et al. and *Zarbock* et al. used web-based randomization to ensure concealed allocation [18, 20]. We further acknowledge the exploratory nature of the NMA, which was facilitated by the absence of direct comparisons. Complete blinding of the intervention was not possible in these RCTs as the medical personnel had to follow the treatment algorithm if randomized to the intervention group. Lack of blinding is an important limitation in all studies and may have potentially impacted some of the outcomes, for instance, timing of RRT. In the PrevAKI-II trial by *Zarbock* et al., endpoint assessment was undertaken by research staff who were not involved in providing anesthesia and perioperative care. Missing or incomplete outcome data in the studies were explained reasonably, and there was no evidence of selective reporting.

Despite these limitations and no direct comparison of the application of AKI care bundles with and without urinary biomarkers, this meta-analysis of the existing literature confirmed the beneficial effect of AKI care bundles on composite renal outcomes and the role of new biomarkers in this setting. A trend of MAKE reduction was depicted using a ranking diagram via NMA, which utilized data from studies that compared each intervention to a common comparator. To the best of our knowledge, this is the most updated meta-analysis in this area. The results of ongoing RCTs comparing biomarker-guided management protocols with standard care in high-risk patients, such as BigpAK-2 and PrevProgAKI, are eagerly awaited (NCT04647396 and NCT05275218) [38, 39].

## Conclusions

Our findings, along with subgroup analysis of RCTs only, strongly suggest a significant trend toward reduced risk of MAKE with the application of AKI care bundles. Furthermore, better outcomes could be observed when kidney biomarkers were incorporated to allow enrichment, enable earlier AKI diagnosis and to guide intervention strategies. Further studies are necessary to explore the impact of individual components of the AKI care bundles and to identify the optimal protocol [30].

### Supplementary Information

**Additional file 1**. Supplementary appendix.

## Data Availability

The datasets used and/or analyzed during the current study are available from the corresponding author on reasonable request.
