# Supplementary material for: Improvement of composite kidney outcomes by AKI care bundles: a systematic review and meta-analysis
Source: Crit Care. 2023 Oct 9;27:390. doi: 10.1186/s13054-023-04641-0 (PMC10563300; doi:10.1186/s13054-023-04641-0)
Supplement: Supplementary file 1 — Additional file 1. Supplementary appendix. [file 13054_2023_4641_MOESM1_ESM.docx]

**Supplementary appendix**

This supplementary appendix provides:

1. Search equation via PubMed, EMBASE and Cochrane library
2. PRISMA abstract checklist. (Supplementary Table 1.)
3. PRISMA checklist. (Supplementary Table 2.)
4. Characteristics of the individual element of the care bundles adopted by each study (Supplementary Table 3.)
5. Trial Sequential Analysis (Supplementary Figure 2a-b.)
6. Risk of bias summary (Supplementary Figure 1a-b.)
7. Funnel plot (Supplementary Figure 3.)
8. Summary of the feature in each study
9. Quality assessment the GRADE results (Supplementary Tables: 9.1-9.10.)
10. Subgroup analyses on cardiovascular surgery (Supplementary Figure 4a-d.)
11. Network meta-analysis (Figure 5)
12. Hierarchical summary receiver operating characteristic (HSROC) curves (Figure 6a-b)
13. **Search equation via PubMed, EMBASE, and Cochrane library**

**Appendix.**

Search strategies for the different databases ran on January 29, 2023.

**Pubmed searching strategy:**

No. Query Results (6781)

("acute kidney injury"[MeSH Terms] OR ("acute"[All Fields] AND "kidney"[All Fields] AND "injury"[All Fields]) OR "acute kidney injury"[All Fields] OR ("acute kidney injury"[MeSH Terms] OR ("acute"[All Fields] AND "kidney"[All Fields] AND "injury"[All Fields]) OR "acute kidney injury"[All Fields] OR ("acute"[All Fields] AND "renal"[All Fields] AND "failure"[All Fields]) OR "acute renal failure"[All Fields]) OR ("acute kidney injury"[MeSH Terms] OR ("acute"[All Fields] AND "kidney"[All Fields] AND "injury"[All Fields]) OR "acute kidney injury"[All Fields] OR ("acute"[All Fields] AND "kidney"[All Fields] AND "failure"[All Fields]) OR "acute kidney failure"[All Fields]) OR (("kidney"[MeSH Terms] OR "kidney"[All Fields] OR "kidneys"[All Fields] OR "kidney s"[All Fields]) AND ("injurie"[All Fields] OR "injuried"[All Fields] OR "injuries"[MeSH Subheading] OR "injuries"[All Fields] OR "wounds and injuries"[MeSH Terms] OR ("wounds"[All Fields] AND "injuries"[All Fields]) OR "wounds and injuries"[All Fields] OR "injurious"[All Fields] OR "injury s"[All Fields] OR "injuryed"[All Fields] OR "injurys"[All Fields] OR "injury"[All Fields])) OR (("renal"[All Fields] OR "renals"[All Fields]) AND ("injurie"[All Fields] OR "injuried"[All Fields] OR "injuries"[MeSH Subheading] OR "injuries"[All Fields] OR "wounds and injuries"[MeSH Terms] OR ("wounds"[All Fields] AND "injuries"[All Fields]) OR "wounds and injuries"[All Fields] OR "injurious"[All Fields] OR "injury s"[All Fields] OR "injuryed"[All Fields] OR "injurys"[All Fields] OR "injury"[All Fields])) OR ("acute kidney injury"[MeSH Terms] OR ("acute"[All Fields] AND "kidney"[All Fields] AND "injury"[All Fields]) OR "acute kidney injury"[All Fields] OR ("acute"[All Fields] AND "renal"[All Fields] AND "insufficiency"[All Fields]) OR "acute renal insufficiency"[All Fields]) OR ("renal insufficiency"[MeSH Terms] OR ("renal"[All Fields] AND "insufficiency"[All Fields]) OR "renal insufficiency"[All Fields])) AND ("prevent"[All Fields] OR "preventability"[All Fields] OR "preventable"[All Fields] OR "preventative"[All Fields] OR "preventatively"[All Fields] OR "preventatives"[All Fields] OR "prevented"[All Fields] OR "preventing"[All Fields] OR "prevention and control"[MeSH Subheading] OR ("prevention"[All Fields] AND "control"[All Fields]) OR "prevention and control"[All Fields] OR "prevention"[All Fields] OR "prevention s"[All Fields] OR "preventions"[All Fields] OR "preventive"[All Fields] OR "preventively"[All Fields] OR "preventives"[All Fields] OR "prevents"[All Fields] OR ("protect"[All Fields] OR "protected"[All Fields] OR "protecting"[All Fields] OR "protection"[All Fields] OR "protections"[All Fields] OR "protective agents"[Pharmacological Action] OR "protective agents"[MeSH Terms] OR ("protective"[All Fields] AND "agents"[All Fields]) OR "protective agents"[All Fields] OR "protectant"[All Fields] OR "protectants"[All Fields] OR "protective"[All Fields] OR "protectively"[All Fields] OR "protectiveness"[All Fields] OR "protectives"[All Fields] OR "protects"[All Fields]) OR ("KDIGO"[All Fields] AND ("patient care bundles"[MeSH Terms] OR ("patient"[All Fields] AND "care"[All Fields] AND "bundles"[All Fields]) OR "patient care bundles"[All Fields] OR ("care"[All Fields] AND "bundle"[All Fields]) OR "care bundle"[All Fields])) OR ("patient care bundles"[MeSH Terms] OR ("patient"[All Fields] AND "care"[All Fields] AND "bundles"[All Fields]) OR "patient care bundles"[All Fields] OR ("care"[All Fields] AND "bundle"[All Fields]) OR "care bundle"[All Fields]) OR ("bundle"[All Fields] OR "bundle s"[All Fields] OR "bundled"[All Fields] OR "bundles"[All Fields] OR "bundling"[All Fields])) AND ("biomarker s"[All Fields] OR "biomarkers"[MeSH Terms] OR "biomarkers"[All Fields] OR "biomarker"[All Fields] OR ("lipocalin 2"[MeSH Terms] OR "lipocalin 2"[All Fields] OR ("neutrophil"[All Fields] AND "gelatinase"[All Fields] AND "associated"[All Fields] AND "lipocalin"[All Fields]) OR "neutrophil gelatinase associated lipocalin"[All Fields]) OR "NGAL"[All Fields] OR (("tissue s"[All Fields] OR "tissues"[MeSH Terms] OR "tissues"[All Fields] OR "tissue"[All Fields]) AND ("antagonists and inhibitors"[MeSH Subheading] OR ("antagonists"[All Fields] AND "inhibitors"[All Fields]) OR "antagonists and inhibitors"[All Fields] OR "inhibitors"[All Fields] OR "inhibitor"[All Fields] OR "inhibitor s"[All Fields]) AND "metalloproteinase-2"[All Fields] AND "x"[All Fields] AND ("insulin like growth factor binding protein related protein 1"[Supplementary Concept] OR "insulin like growth factor binding protein related protein 1"[All Fields] OR "insulin like growth factor binding protein 7"[All Fields])) OR "TIMP-2IGFBP7"[All Fields] OR ("cell cycle"[MeSH Terms] OR ("cell"[All Fields] AND "cycle"[All Fields]) OR "cell cycle"[All Fields]))

**EMBASE**

No. Query Results (112)

('acute kidney injury'/exp OR 'acute kidney injury' OR (acute AND ('kidney'/exp OR kidney) AND ('injury'/exp OR injury)) OR biomarker OR 'timp2 igfbp7' OR 'neutrophil gelatinase associated lipocalin') AND 'care bundle'

**Cochrane searching strategy:**

No. Query Results (344)

(acute kidney injury OR acute renal failure OR acute kidney failure OR acute renal insufficiency OR renal insufficiency) in ALL TEXT AND (Prevention OR Protection OR KDIGO care bundle OR care bundle) in ALL TEXT AND (Biomarker OR NGAL OR neutrophil gelatinase-associated lipocalin OR TIMP-2・IGFBP7 OR cell cycle OR tissue inhibitor metalloproteinase-2 x insulin-like growth factor-binding protein 7) in ALL TEXT

1. **Supplementary Table 1. PRISMA abstract checklist**

| **Section and Topic** | **Item #** | **Checklist item** | **Reported (Yes/No)** |
| --- | --- | --- | --- |
| **TITLE** | | |  |
| Title | 1 | Identify the report as a systematic review. | Yes |
| **BACKGROUND** | | |  |
| Objectives | 2 | Provide an explicit statement of the main objective(s) or question(s) the review addresses. | Yes |
| **METHODS** | | |  |
| Eligibility criteria | 3 | Specify the inclusion and exclusion criteria for the review. | Yes |
| Information sources | 4 | Specify the information sources (e.g. databases, registers) used to identify studies and the date when each was last searched. | Not in abstract |
| Risk of bias | 5 | Specify the methods used to assess risk of bias in the included studies. | Not in abstract |
| Synthesis of results | 6 | Specify the methods used to present and synthesise results. | Not in abstract |
| **RESULTS** | | |  |
| Included studies | 7 | Give the total number of included studies and participants and summarise relevant characteristics of studies. | Yes |
| Synthesis of results | 8 | Present results for main outcomes, preferably indicating the number of included studies and participants for each. If meta-analysis was done, report the summary estimate and confidence/credible interval. If comparing groups, indicate the direction of the effect (i.e. which group is favoured). | Yes |
| **DISCUSSION** | | |  |
| Limitations of evidence | 9 | Provide a brief summary of the limitations of the evidence included in the review (e.g. study risk of bias, inconsistency and imprecision). | Not in abstract |
| Interpretation | 10 | Provide a general interpretation of the results and important implications. | Yes |
| **OTHER** | | |  |
| Funding | 11 | Specify the primary source of funding for the review. | Not in abstract |
| Registration | 12 | Provide the register name and registration number. | No |

1. **Supplementary Table 2. PRISMA checklist**

| **Section and Topic** | **Item #** | **Checklist item** | **Location where item is reported** |
| --- | --- | --- | --- |
| **TITLE** | | |  |
| Title | 1 | Identify the report as a systematic review. | Page 1 |
| **ABSTRACT** | | |  |
| Abstract | 2 | See the PRISMA 2020 for Abstracts checklist. | Page 4-5 |
| **INTRODUCTION** | | |  |
| Rationale | 3 | Describe the rationale for the review in the context of existing knowledge. | Page 8-9 |
| Objectives | 4 | Provide an explicit statement of the objective(s) or question(s) the review addresses. | Page 9 |
| **METHODS** | | |  |
| Eligibility criteria | 5 | Specify the inclusion and exclusion criteria for the review and how studies were grouped for the syntheses. | Page 10 |
| Information sources | 6 | Specify all databases, registers, websites, organisations, reference lists and other sources searched or consulted to identify studies. Specify the date when each source was last searched or consulted. | Page 9 |
| Search strategy | 7 | Present the full search strategies for all databases, registers and websites, including any filters and limits used. | Page 9-10 |
| Selection process | 8 | Specify the methods used to decide whether a study met the inclusion criteria of the review, including how many reviewers screened each record and each report retrieved, whether they worked independently, and if applicable, details of automation tools used in the process. | Page 10-11 |
| Data collection process | 9 | Specify the methods used to collect data from reports, including how many reviewers collected data from each report, whether they worked independently, any processes for obtaining or confirming data from study investigators, and if applicable, details of automation tools used in the process. | Page 10-11 |
| Data items | 10a | List and define all outcomes for which data were sought. Specify whether all results that were compatible with each outcome domain in each study were sought (e.g. for all measures, time points, analyses), and if not, the methods used to decide which results to collect. | Page 11 |
|  | 10b | List and define all other variables for which data were sought (e.g. participant and intervention characteristics, funding sources). Describe any assumptions made about any missing or unclear information. | Page 10 |
| Study risk of bias assessment | 11 | Specify the methods used to assess risk of bias in the included studies, including details of the tool(s) used, how many reviewers assessed each study and whether they worked independently, and if applicable, details of automation tools used in the process. | Page 11,12 |
| Effect measures | 12 | Specify for each outcome the effect measure(s) (e.g. risk ratio, mean difference) used in the synthesis or presentation of results. | Page 11 |
| Synthesis methods | 13a | Describe the processes used to decide which studies were eligible for each synthesis (e.g. tabulating the study intervention characteristics and comparing against the planned groups for each synthesis (item #5)). | Page 11 |
|  | 13b | Describe any methods required to prepare the data for presentation or synthesis, such as handling of missing summary statistics, or data conversions. | Page 11 |
|  | 13c | Describe any methods used to tabulate or visually display results of individual studies and syntheses. | Page 11 |
|  | 13d | Describe any methods used to synthesize results and provide a rationale for the choice(s). If meta-analysis was performed, describe the model(s), method(s) to identify the presence and extent of statistical heterogeneity, and software package(s) used. | Page 12 |
|  | 13e | Describe any methods used to explore possible causes of heterogeneity among study results (e.g. subgroup analysis, meta-regression). | Page 11 |
|  | 13f | Describe any sensitivity analyses conducted to assess robustness of the synthesized results. | Page 11 |
| Reporting bias assessment | 14 | Describe any methods used to assess risk of bias due to missing results in a synthesis (arising from reporting biases). | Page 11 |
| Certainty assessment | 15 | Describe any methods used to assess certainty (or confidence) in the body of evidence for an outcome. | Page 12 |
| **RESULTS** | | |  |
| Study selection | 16a | Describe the results of the search and selection process, from the number of records identified in the search to the number of studies included in the review, ideally using a flow diagram. | Page 12,13, Fig 1 |
|  | 16b | Cite studies that might appear to meet the inclusion criteria, but which were excluded, and explain why they were excluded. | Page 12,13, Fig 1 |
| Study characteristics | 17 | Cite each included study and present its characteristics. | Table 1, Supplementary appendix 7 |
| Risk of bias in studies | 18 | Present assessments of risk of bias for each included study. | Page 14,15,  Supplementary appendix 5,6,8 |
| Results of individual studies | 19 | For all outcomes, present, for each study: (a) summary statistics for each group (where appropriate) and (b) an effect estimate and its precision (e.g. confidence/credible interval), ideally using structured tables or plots. | Figure 2 |
| Results of syntheses | 20a | For each synthesis, briefly summarise the characteristics and risk of bias among contributing studies. | Page 14,15, Supplementary appendix 5 |
|  | 20b | Present results of all statistical syntheses conducted. If meta-analysis was done, present for each the summary estimate and its precision (e.g. confidence/credible interval) and measures of statistical heterogeneity. If comparing groups, describe the direction of the effect. | Page 16,17,18, Fig 2 |
|  | 20c | Present results of all investigations of possible causes of heterogeneity among study results. | Page 18, supplementary appendix 6 |
|  | 20d | Present results of all sensitivity analyses conducted to assess the robustness of the synthesized results. | Page 18 |
| Reporting biases | 21 | Present assessments of risk of bias due to missing results (arising from reporting biases) for each synthesis assessed. | Page 14, Supplementary appendix 5 |
| Certainty of evidence | 22 | Present assessments of certainty (or confidence) in the body of evidence for each outcome assessed. | Page 18, Supplementary appendix 8 |
| **DISCUSSION** | | |  |
| Discussion | 23a | Provide a general interpretation of the results in the context of other evidence. | Page 19-21 |
|  | 23b | Discuss any limitations of the evidence included in the review. | Page 21,22 |
|  | 23c | Discuss any limitations of the review processes used. | Page 21,22 |
|  | 23d | Discuss implications of the results for practice, policy, and future research. | Page 23 |
| **OTHER INFORMATION** | | |  |
| Registration and protocol | 24a | Provide registration information for the review, including register name and registration number, or state that the review was not registered. | Page |
|  | 24b | Indicate where the review protocol can be accessed, or state that a protocol was not prepared. | Page 9,10 |
|  | 24c | Describe and explain any amendments to information provided at registration or in the protocol. | Page |
| Support | 25 | Describe sources of financial or non-financial support for the review, and the role of the funders or sponsors in the review. | Page 24 |
| Competing interests | 26 | Declare any competing interests of review authors. | Page 25 |
| Availability of data, code and other materials | 27 | Report which of the following are publicly available and where they can be found: template data collection forms; data extracted from included studies; data used for all analyses; analytic code; any other materials used in the review. | Page 24 |

1. **Supplementary Table 3. Characteristics of the individual element of the care bundles adopted by each study**

| **No** | **Study**  **(year)** | **Nephrotoxic drugs and radiocontrast** | **Volume status and perfusion pressure** | **Hemodynamic monitoring** | **Monitor serum creatinine and urine output** | **Avoid hyperglycemia** | **Others** |
| --- | --- | --- | --- | --- | --- | --- | --- |
| 1 | Kolhe et al. 2015 (1) | 52.9% of patients had their medications checked for nephrotoxins and 21.2% of patients were found to have nephrotoxic medications. | Monitoring fluid balance, blood tests and early warning score were instituted in 70.9% of patients | NA | Monitoring fluid balance, blood tests and early warning score were instituted in 70.9% of patients | NA | Electronic recognition and alerting system  Nephrology advice was obtained in 15.7% of patients and only 1.3% of doctors documented that they had referred to AKI guidelines on hospital website. |
| 2 | Kolhe et al. 2016 (2) | 34.8% of patients had nephrotoxins in their medication list. | Monitoring of fluid volume, blood tests and early warning score were assessed to be required in 62% of patients. | NA | Monitoring of fluid volume, blood tests and early warning score were assessed to be required in 62% of patients. | NA | AKI care bundle derived from National Confidential Enquiry into Patient Outcome and Death (NCEPOD)  Nephrology advice was obtained in 14.5% of patients and only 2.4% of doctors documented that they had referred to AKI guidelines on hospital website. |
| 3 | Meersch et al. 2017 (3) | Avoidance of nephrotoxic agents, discontinuation of ACEi and ARBs for the first 48 hours after surgery, consideration of alternatives to radiocontrast agent | Close hemodynamic monitoring by using a PICCO catheter with an optimization of the volume status and hemodynamic parameters | Close hemodynamic monitoring by using a PICCO catheter with an optimization of the volume status and hemodynamic parameters | Close monitoring of serum creatinine and urine output | Avoidance of hyperglycemia for the first 72 hours after surgery | KDIGO cardiothoracic surgery bundle |
| 4 | Göcze et al. 2018 (4) | Recommendations  on current potentially nephrotoxic medications were given for  21 patients (35%). | Fluid balance was monitor on day 1 and 2 | optimization of hemodynamics | Changes of serum creatinine and urine output were monitored | NA | KDIGO bundle  All patients in the intervention group had undergone nephrology  consultation before the start of any intervention. |
| 5 | Kapoor et al. 2019 (5) | No nephrotoxic drugs were  administered in the peri-operative course of management. | All patients received maintenance fluid ringer lactate 1 ml/  kg/h. | Central venous pressure was maintained at 6–  8 mmHg; MAP > 90 mmHg; fluid and inotropes use were mentioned | Hourly urine output was monitored | The blood glucose  level was maintained in between 80 and 180 mg/dL. | Goal-directed therapy |
| 6 | Schanz et al. 2019 (6) | Avoidance/stopping of nephrotoxic drugs | Optimization of haemodynamics and fluid status | Optimization of haemodynamics and fluid status | Monitoring serum creatinine and urine output on admission, day 1-3, and at discharge for serum creatinine | Preventing  hyperglycaemia; hyperglycaemia was defined as a blood glucose level > 150 mg/dL for > 3 hours | KDIGO bundle |
| 7 | Engelman et al. 2020 (7) | Avoidance of nephrotoxins | Targeted goal-directed fluid management | Continued invasive hemodynamic monitoring  and its optimization | continued close monitoring of urinary output via  indwelling urinary catheters | Avoidance  of hyperglycemia for the first 72 hours after surgery | KDIGO bundle |
| 8 | Koeze et al. 2020 (8) | Critical evaluation of the indication and dose of nephrotoxic drugs | Optimizing fluid balance (based on urine output, serum lactate levels and/or central venous oxygen saturation), discontinuation of diuretics | Maintaining a MAP of at least 65 mmHg with potential use of vasopressors | Monitor urine output for optimization of fluid balance | NA | “Save the kidney” educational intervention bundle was encouraged in all patients |
| 9 | Zarbock et al. 2021 (9) | Avoidance of nephrotoxic agents, discontinuation  of ACEi and ARB  during the first 48 hours after surgery, avoidance of hydroxyethyl starch, gelatine, and chloride-rich  solutions (including 0.9% saline), consideration  of alternatives to radiocontrast agents | Close hemodynamic monitoring by using a functional  hemodynamic monitoring with an optimization of  the volume status, and hemodynamic parameters | Close hemodynamic monitoring by using a functional  hemodynamic monitoring with an optimization of  the volume status, and hemodynamic parameters | Close monitoring  of serum creatinine (every 12 hours), fluid balance  and urine output (hourly) | Avoidance of hyperglycemia  in the first 72 hours after surgery (defined as  blood glucose levels >150 mg/dL for >3 hours) | KDIGO bundle |
| 10 | Halmy et al. 2021 (10) | No NSAIDs or ACEi/ARBs for at least 48 hours, daily adjust drugs to patient’s GFR, avoid vancomycin, if needed adjust doses and check target level | Volume status was assessed with static  or dynamic tests such as central venous pressure (CVP) measurement, echocardiography,  sonography of inferior vena cava (IVC), or the leg-raising test  Adequate perfusion pressure  was maintained with fluid therapy or vasopressors | Patients with a high risk required  maintenance of mean arterial pressure within a 10%  margin of preoperative values, and extended hemodynamic monitoring. | Daily creatinine measurements, hourly monitoring of urine output | NA | KDIGO bundle  Patients with a high risk required  additional nephrology consultation |
| 11 | Couturier et al. 2021 (11) | NSAIDs were contraindicated; renin angiotensin blockers were introduced after 72 hours | Fluid therapy and diuretic were recorded | According to the  hemodynamic monitoring and the transthoracic cardiac echography, fluid, noradrenalin or dobutamine were used to obtain MAP > 65 mmHg, a venous oxygen saturation *>*65%, a cardiac frequency  between 65 and 100 bpm and a variation of pulse pressure *<*13 | NA | NA | KDIGO bundle |
| 12 | Bourdeaux et al. 2021 (12) | Medication | Fluid input/output | NA | Urine output, creatinine | NA | KDIGO bundle |
| 13 | Kotwal et al. 2022 (13) | 25% in the intervention group stopped nephrotoxins, while only 18% in the control group stopped nephrotoxins | NA | NA | NA | NA | STOP AKI management guideline |

Abbreviations: ACEi, angiotensin-converting enzyme inhibitor; ARB, angiotensin-II receptor blocker; MAP, mean arterial pressure; NSAIDs, Non-steroidal anti-inflammatory drugs; NA, not applicable

1. **Supplementary Figure 2a. Trial Sequential Analysis (TSA) of all 13 included studies**


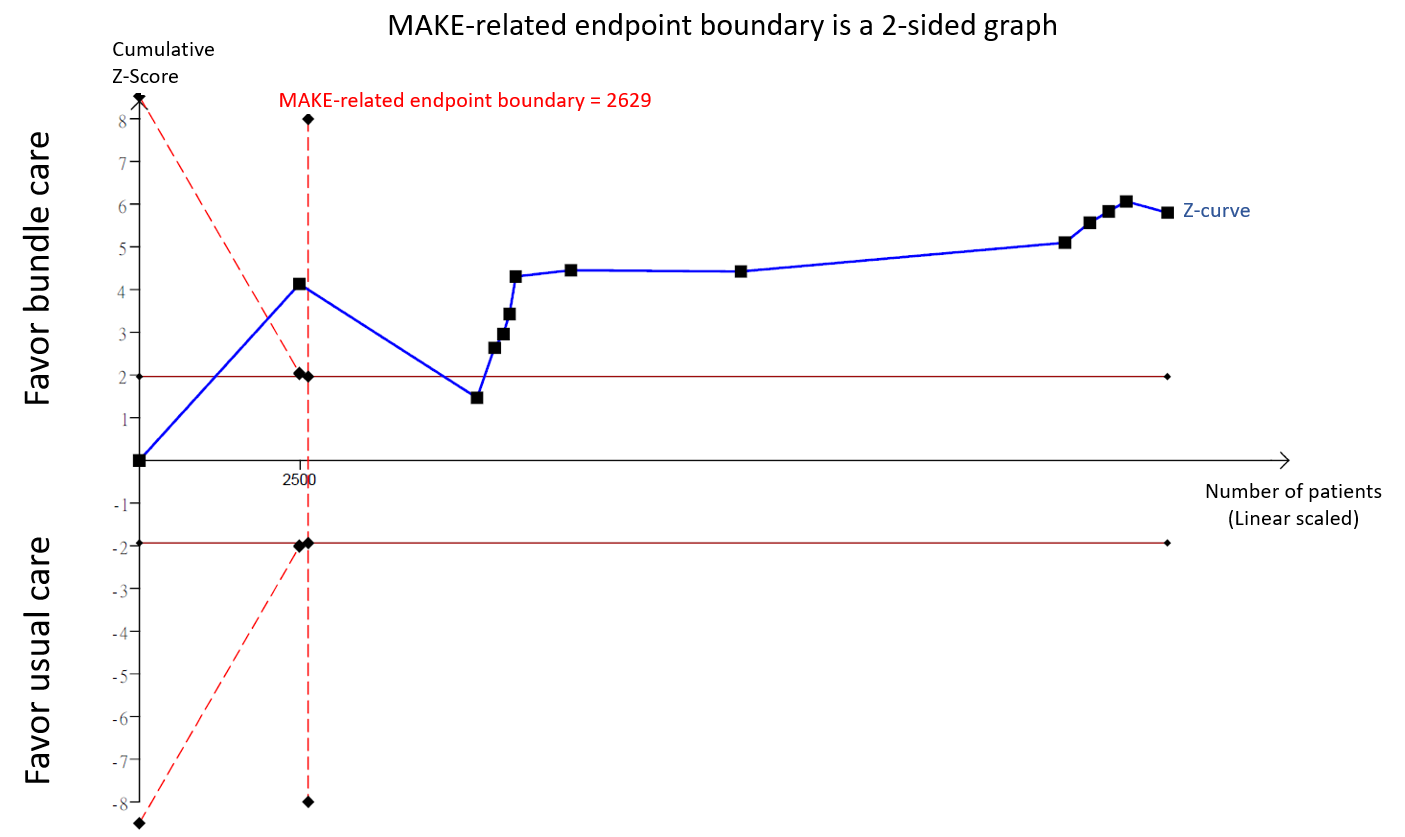


**Supplementary Figure 2b. Trial Sequential Analysis (TSA) if only randomized controlled trials are included**


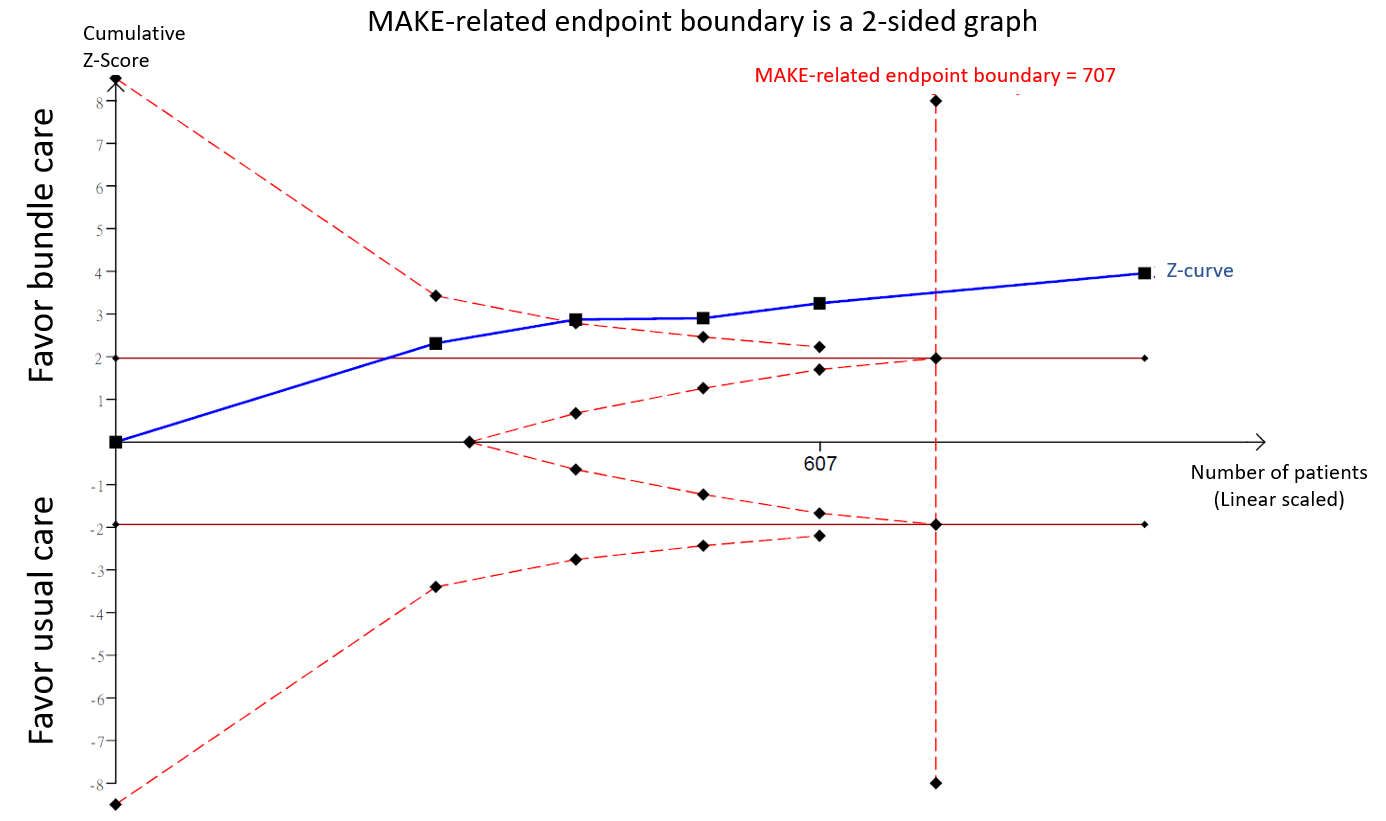


**Abbreviations:** TSA, the significance of AKI care bundles was confirmed as the cumulative z-curve line surpassed the conventional boundary of benefits.

1. **Supplementary Figure 1a. Summary of risk of bias assessment using version 2 of Cochrane risk-of-bias tool for randomized trials (RoB 2)**


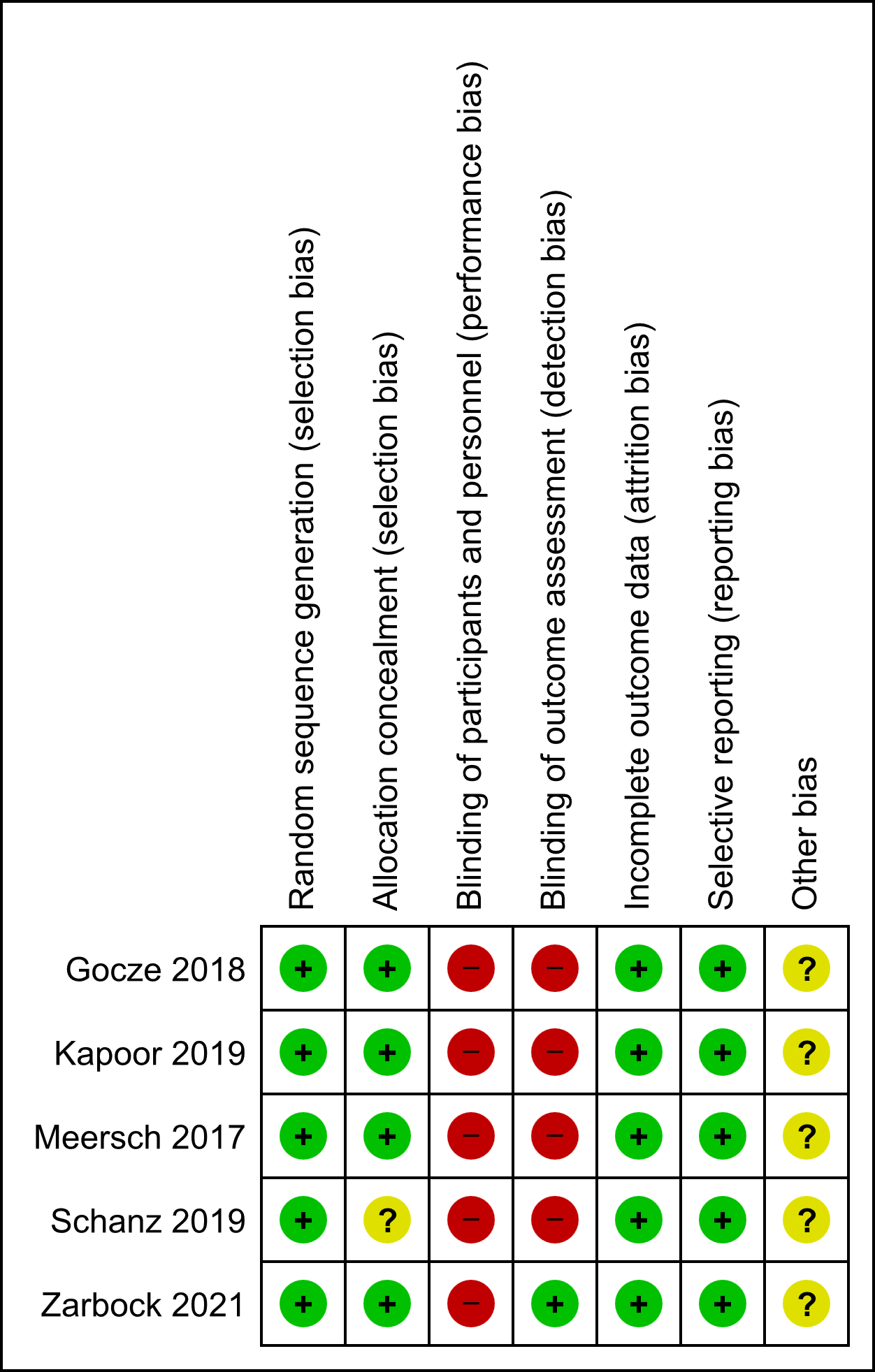


**Supplementary Figure 1b. Summary of risk of bias assessment using Newcastle-Ottawa Quality Assessment Score for cohort studies**


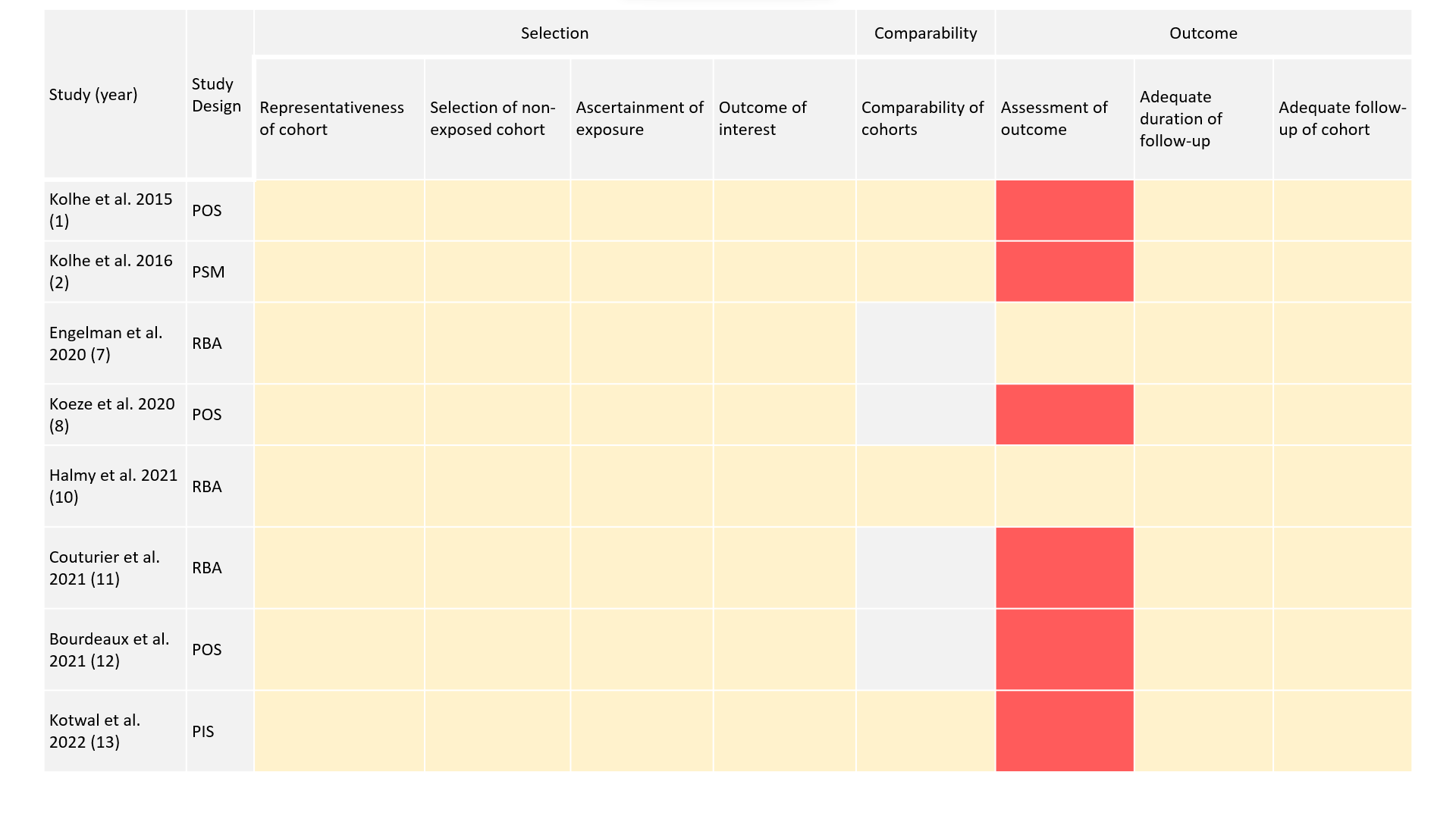


Eight questions were included in the Newcastle-Ottawa Quality Assessment: (1) was the cohort truly* or somewhat* representative of the average in the desirable study population?; (2) was the non-exposed cohort drawn from the same* community as the exposed cohort or a different source?; (3) was the ascertainment of exposure from a secure record*, structured interview*, or written self-report?; (4) was the outcome of interest present at start of study*?; (5) was the study controls for age, sex and marital status*, or other factors*?; (6) was the assessment of outcome by independent blind assessment*, record linkage*, or self-report?; (7) was follow-up long enough for outcomes to occur?; (8) was the follow-up of cohort complete for all subjects*, only less than 20% of subjects lost to follow-up*, or follow-up rate <80% and no description of those lost? *Asterisk represents characteristic with lower risk of bias. Yellow boxes represent low level of risk of bias. Red boxes represent high level of risk of bias. White boxes represent unclear risk of bias.

**Abbreviations:** PIS, prospective interventional study; POS, prospective observation study; PSM, propensity score-matched cohort study; RBA, retrospective before-after study.

1. **Supplementary Figure 3. Funnel plot**

**
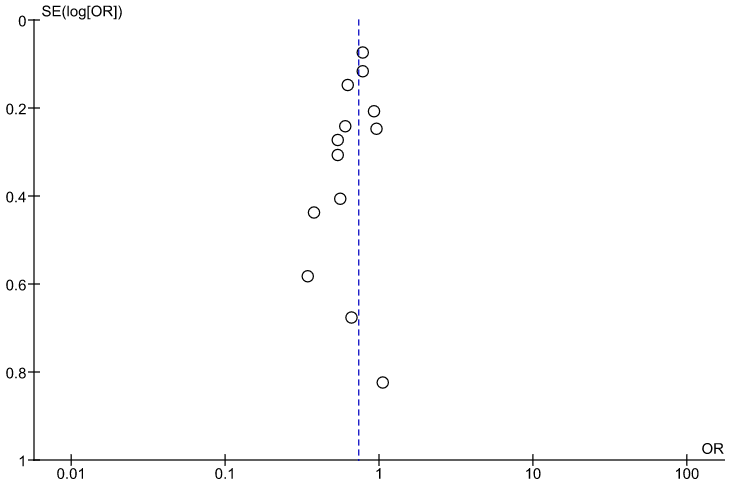
**

1. **Summary of relevant characteristics of included study**

In our study, a total 16 articles were included in this meta-analysis.

**For analysis of the clinical use of KDIGO bundle, (n = 15) were included.**

Meersch et al. (2017) included 276 patients undergoing cardiac surgery, and found that “KDIGO bundle” consisting of optimization of volume status and hemodynamics, avoidance of nephrotoxic drugs, and preventing hyperglycemia in high risk patients defined as urinary [TIMP-2]·[IGFBP7] > 0.3.

AKI was significantly reduced with the intervention compared to controls [55.1 vs. 71.7%; ARR 16.6% (95 CI5.5–27.9%); p = 0.004]

Go¨cze et al. (2018) included 121 patients undergoing cardiac surgery, “KDIGO bundle” consisting of early optimization of fluid status, maintenance of perfusion pressure, discontinuation of nephrotoxic agents in increased AKI risk after major abdominal surgery that was determined by urinary [TIMP-2]·[IGFBP7] > 0.3.

Subgroup analysis of patients with [TIMP-2]·[IGFBP7] 0.3 to 2.0 demonstrated a significantly reduced incidence of AKI 13/48 (27.1%) in the intervention group compared to control 24/50 (48.0%, P . 0.03). Incidence of moderate and severe AKI (P . 0.04), incidence of creatinine increase >25% of baseline value (P . 0.01), length of ICU, and hospital stay (P . 0.04) were significantly lower in the intervention group. Intervention was also associated with cost reduction.

Schanz et al. (2018) included 100 patients in the emergency department, intervention included one-time nephrological and KDIGO 2012 recommendations on AKI in increased AKI risk after major abdominal surgery that was determined by urinary [TIMP-2]·[IGFBP7] >0.3. The intervention group had significantly (P<0.05) lower serum creatinine (SCr) on Day 2 and lower maximum SCr and tended (P.0.08) to have higher urine output (UOP) at Day 3 than the non-intervention group. No patient in the intervention group needed RRT (0 versus 3) during the hospital stay (P.0.09).

Engelman et al. (2020) included 847 patients undergoing cardiac surgery, urinary biomarkers (UB) that was [TIMP2] ·[IGFBP7] ≥ 0.3 triggered activation of KDIGO cardiac surgery bundle (predefined staged protocol, including targeted goal-directed fluid management, liberalized transfusion thresholds, continued invasive hemodynamic monitoring and its optimization in the intensive care unit, and avoidance of nephrotoxins.)

The incidence of stage 2/3 AKI was compared in 435 pre-UB patients and 412 post-UB patients. Fifty-five percent of the post-UB patients had a moderate or high UB score ≥ 0.3. Ten patients (2.30%) had stage 2/3 AKI pre-UB, compared with 1 patient (0.24%) post-UB, a relative reduction of 89% (P = 0.01). The negative predictive value for AKI of UB<0.3 was 100%.

Kane-Gill et al. (2020) included 100 patients an intensive care unit, “KDIGO bundle” was implemented in response to [TIMP2]•[IGFBP7] test results.

Sixty-one patients had [TIMP2]•[IGFBP7] >0.3 and 46 (75.4%) of these patients received at least one strategy consistent with KDIGO. By contrast, nine patients (23.1%) with [TIMP2]•[IGFBP7] ≤0.3 received one or more components of the KDIGO bundle (p < .001).

Zarbock et al. (2021) included 278 patients undergoing cardiac surgery, “KDIGO bundle” consisting of optimization of volume status and hemodynamics, functional hemodynamic monitoring, avoidance of nephrotoxic drugs, and prevention of hyperglycemia in high-risk patients identified by [TIMP-2]•[IGFBP7] ≥0.3

In the intervention group, 65.4% of patients received the complete bundle as compared to 4.2% in the control group (ARR 61.2 [95% CI, 52.6-69.9]; P < .001). AKI rates were statistically not different in both groups. However, the occurrence of moderate and severe AKI was significantly lower in the intervention group as compared to the control group (14.0% vs 23.9%; ARR 10.0% [95% CI, 0.9-19.1]; P = .034).

Gocze et al. (2021) included 294 patients undergoing major noncardiac surgery, biomarker-guided kidney-protection intervention. Patients with moderate risk and high risk received KDIGO bundle.

Protocol implementation significantly increased the recovery of AKI (36/46, 78% compared to control 27/48, 56%, (p = 0.025)) and reduced the length of the ICU stay (p < 0.001). 46/143 (32%) and 12/151 (8%) in the protocol implementation group compared to 48/151 (32%) and 18/151 (12%) in the historical control group. Patients with AKI reversal within the first 7 postoperative days had lower in-hospital mortality than patients without AKI reversal.

Couturier et al. (2021) included 382 patients undergoing major cardiac surgery, The patients with high AKI risk were defined by a urinary [TIMP-2]•[IGFBP7] >0.3 and received specific renal supportive measures according to an ICU local protocol based on KDIGO guidelines.

The incidence of AKI was significantly reduced in the [TIMP-2]•[IGFBP7] cohort (20.5 vs 29.9%, p < 0.05). In multivariate analysis, patients of the [TIMP-2]•[IGFBP7] cohort had a lower risk of developing AKI (p = 0.029).

Kolhe et al. (2015) included 2297 hospitalized patients who had 2500 AKI episodes, with 1209 and 1291 episodes occurring before and after implementation of the of the AKI care bundle (CB). In-hospital case-fatality was significantly lower in the early CB group (18% versus 23.1%, p 0.046). Progression to higher AKI stages was lower in the early CB group (3.9% vs. 8.1%, p 0.01). In multivariate analysis, patients in the early CB group had lower odds of death at discharge (0.641; 95% CI 0.46, 0.891), 30 days (0.707; 95% CI 0.527, 0.950), 60 days (0.704; 95% CI 0.526, 0.941) and after a median of 134 days (0.771; 95% CI 0.62, 0.958).

Kolhe et al. (2016) included 3518 hospitalized patients who were admitted with or developed AKI. The AKI care bundle (CB) was completed in 25.6% of patients. The unadjusted case–fatality was higher when the AKICB was not completed versus when the AKI-CB was completed (24.4 versus 20.4%, P = 0.017). In multivariable analysis, AKICB completion within 24 h was associated with lower odds for in-hospital death [OR: 0.76; 95% CI: 0.62–0.92]. Increasing age (OR: 1.04; 95% CI: 1.03–1.05), hospital-acquired AKI (OR: 1.28; 95% CI: 1.04–1.58), AKI stage 2 (OR: 1.91; 95% CI: 1.53–2.39) and increasing Charlson’s comorbidity index (CCI) [OR: 3.31 (95% CI: 2.37–4.64) for CCI of more than 5 compared with zero] had higher odds for death, whereas AKI during elective admission was associated with lower odds for death (OR: 0.29; 95% CI: 0.16–0.52). Progression to higher AKI stages was lower when the AKI-CB was completed (4.2 versus 6.7%, P = 0.02).

Sykes et al. (2018) included all the population in the Salford Royal NHS Foundation Trust (SRFT).

On 10 Collaborative wards, development of an AKI care bundle via multidisciplinary team (MDT) plan, do. Results showed a 15.6% reduction in hospitalwide-acquired AKI, with a 22.3% reduction on the

collaborative wards. Trust-wide rates of progression of AKI 1 to AKI 2 or 3 showed normal variation, whereas there was a 48.5% reduction in AKI progression on the Collaborative wards.

Selby et al. (2019) included 24,059 AKI episodes in hospitalized patients. An AKI electronic detection and alerting system, an AKI care bundle, an educational program was implemented.

An overall 30-day mortality of 24.5%, with no difference between control and intervention periods. Hospital length of stay was reduced with the intervention (decreases of 0.7, 1.1, and 1.3 days at the 0.5, 0.6, and 0.7 quantiles, respectively). AKI incidence increased and was mirrored by an increase in the proportion of patients with a coded diagnosis of AKI. 1048 patients showed improvements in several metrics including AKI recognition, medication optimization, and fluid assessment.

Koeze et al. (2020) included 2642 ICU patients, A ‘Save the Kidney’ (STK) consisted of optimizing the fluid balance (based on urine output, serum lactate levels and/or central venous oxygen saturation), discontinuation of diuretics, maintaining a mean arterial pressure of at least 65 mmHg with the potential use of vasopressors and critical evaluation of the indication and dose of nephrotoxic drugs.

The primary outcome was the composite of mortality, renal replacement therapy (RRT), and progression of AKI. Secondary outcomes were the components of the composite outcome the severity of AKI, ICU length of stay and in-hospital mortality.

The primary outcome (composite of mortality, renal replacement therapy (RRT), and progression of AKI) occurred in 451 patients (33%) in the STK group versus 375 patients (29%) in the usual care group, RR 1.16, 95% CI 1.03–1.3, p < 0.001. ICU mortality in 6.8% versus 5.6%, (RR 1.22, 95% CI 0.90–1.64, p = 0.068), RRT in 1.6% versus 3.6% (RR 0.46, 95% CI 0.28–0.76, p = 0.002), and AKI progression in 28% versus 24% (RR 1.18, 95% CI 1.04–1.35, p = 0.001).

Bourdeaux et al. (2021) included 5044 ICU patients, intervention consisted of the AKIN guidelines and AKI care bundle which included guidance for medication usage.

The proportion of patients worsening from Stage 1 AKI decreased from 42% (control) to 33.5% (intervention), p = 0.002. The proportion of incorrect enoxaparin doses decreased from 1.72% (control) to 0.6% (intervention), p < 0.001. The prevalence of any AKI decreased from 43.1% (control) to 37.5% (intervention), p < 0.05.

Endre et al. (2022) included 639 patients undergoing cardiac surgery,

The intervention bundle included an interruptive automated alerts (aAlerts) showing AKI stage and baseline creatinine in the eMR, a management guide and junior medical staff education.

Documentation of AKI was better in the intervention group (94.8% versus 83.4%; P=0.001), with higher rates of nephrology consultation (25% versus 19%; P = 0.04) and cessation of nephrotoxins (25.3 versus 18.8%; P=0.045). There was no difference in mortality between intervention versus controls (11.7% versus 13.0%; P = 0.71).

**Information related to type of biomarker type used in different studies**

**For analysis of the clinical use of insulin-like growth factor-binding protein 7 (IGFBP7) and tissue inhibitor of metalloproteinases-2 (TIMP-2) as a marker for acute kidney injury (AKI) risk (n= 8).**

Meersch et al. (2017) included 276 patients undergoing cardiac surgery, and found that “KDIGO bundle” in high risk patients defined as urinary [TIMP-2]·[IGFBP7] > 0.3. AKI was significantly reduced with the intervention compared to controls [55.1 vs. 71.7%; ARR 16.6% (95 CI5.5–27.9%); p = 0.004]

Go¨cze et al. (2018) included 121 patients undergoing cardiac surgery, “KDIGO bundle” in increased AKI risk after major abdominal surgery that was determined by urinary [TIMP-2]·[IGFBP7] > 0.3.

Subgroup analysis of patients with [TIMP-2]·[IGFBP7] 0.3 to 2.0 demonstrated a significantly reduced incidence of AKI 13/48 (27.1%) in the intervention group compared to control 24/50 (48.0%, P . 0.03). Incidence of moderate and severe AKI (P . 0.04), incidence of creatinine increase >25% of baseline value (P . 0.01), length of ICU, and hospital stay (P . 0.04) were significantly lower in the intervention group. Intervention was also associated with cost reduction.

Schanz et al. (2018) included 100 patients in the emergency department, intervention in increased AKI risk after major abdominal surgery that was determined by urinary [TIMP-2]·[IGFBP7] >0.3. The intervention group had significantly (P<0.05) lower serum creatinine (SCr) on Day 2 and lower maximum SCr and tended (P.0.08) to have higher urine output (UOP) at Day 3 than the non-intervention group. No patient in the intervention group needed RRT (0 versus 3) during the hospital stay (P.0.09).

Engelman et al. (2020) included 847 patients undergoing cardiac surgery, urinary biomarkers (UB) that was [TIMP2] ·[IGFBP7] ≥ 0.3 triggered activation of KDIGO cardiac surgery bundle.

The incidence of stage 2/3 AKI was compared in 435 pre-UB patients and 412 post-UB patients. Fifty-five percent of the post-UB patients had a moderate or high UB score ≥ 0.3. Ten patients (2.30%) had stage 2/3 AKI pre-UB, compared with 1 patient (0.24%) post-UB, a relative reduction of 89% (P = 0.01). The negative predictive value for AKI of UB<0.3 was 100%.

Kane-Gill et al. (2020) included 100 patients an intensive care unit, “KDIGO bundle” was implemented in response to [TIMP2]•[IGFBP7] test results.

Sixty-one patients had [TIMP2]•[IGFBP7] >0.3 and 46 (75.4%) of these patients received at least one strategy consistent with KDIGO. By contrast, nine patients (23.1%) with [TIMP2]•[IGFBP7] ≤0.3 received one or more components of the KDIGO bundle (p < .001).

Zarbock et al. (2021) included 278 patients undergoing cardiac surgery, “KDIGO bundle” in high-risk patients identified by [TIMP-2]•[IGFBP7] ≥0.3

In the intervention group, 65.4% of patients received the complete bundle as compared to 4.2% in the control group (ARR 61.2 [95% CI, 52.6-69.9]; P < .001). AKI rates were statistically not different in both groups. However, the occurrence of moderate and severe AKI was significantly lower in the intervention group as compared to the control group (14.0% vs 23.9%; ARR 10.0% [95% CI, 0.9-19.1]; P = .034).

Go¨cze et al. (2021) included 294 patients undergoing major noncardiac surgery, biomarker-guided kidney-protection intervention. Low risk with [TIMP-2]•[IGFBP7] < 0.3; moderate risk [TIMP-2]•[IGFBP7] 0.3–2.0, and high risk [TIMP-2]•[IGFBP7] > 2.0. Patients with moderate risk and high risk received KDIGO bundle.

Protocol implementation significantly increased the recovery of AKI (36/46, 78% compared to control 27/48, 56%, (p = 0.025)) and reduced the length of the ICU stay (p < 0.001). 46/143 (32%) and 12/151 (8%) in the protocol implementation group compared to 48/151 (32%) and 18/151 (12%) in the historical control group. Patients with AKI reversal within the first 7 postoperative days had lower in-hospital mortality than patients without AKI reversal.

Claisse et al. (2021) included 382 patients undergoing major cardiac surgery, The patients with high AKI risk were defined by a urinary [TIMP-2]•[IGFBP7] >0.3 and received specific renal supportive measures according to an ICU local protocol based on KDIGO guidelines.

The incidence of AKI was significantly reduced in the [TIMP-2]•[IGFBP7] cohort (20.5 vs 29.9%, p < 0.05). In multivariate analysis, patients of the [TIMP-2]•[IGFBP7] cohort had a lower risk of developing AKI (p = 0.029).

**For analysis of the clinical use of neutrophil gelatinase associated lipocalin (NGAL) as a biomarker for acute kidney injury (AKI), one study exploring both urine NGAL and plasma NGAL was included.**

Kapoor et al. (2018) included 110 patients comparing plasma (P) and urinary (U)-NGAL levels following the use of GDT versus conventional haemodynamic therapy (CT) in patients major cardiac surgery, and found that U-NGAL was significantly lower immediately post-surgery (T1) in GDT group (25.11 ± 1.5 versus 27.80 ± 1.7 μg/L; p < 0.001) and at 4 h (T2) (38.19 ± 23.6 versus 52.30 ± 28.3 μg/L; p = 0.006) and at 24 h post-operatively (T3) (34.85 ± 14 versus 39.7 ± 11.1 μg/L; p = 0.047). P-NGAL was comparable between groups at T1 but lower in the GDT group at T2 (92.81 ± 4.8 versus 94.77 ± 4.5 μg/L; p = 0.03) and T3 (67.44 ± 3.7 versus 75.96 ± 5.3 μg/L; p < 0.001). U-NGAL levels correlated well with the peak post-operative creatinine as compared to P-NGAL. On-pump patients manifest neutrophil activation, accounting for comparable levels of P-NGAL in the two groups at T1. GDT-based haemodynamic management resulted in lower U-NGAL levels at T1, T2 and T3 and lower P-NGAL levels at T2 and T3.

1. **Assessing quality of evidence according to GRADE**

**9.1. Major Adverse Kidney Events (MAKE)**

| **Certainty assessment** | | | | | | | **№ of patients** | | **Effect** | | **Certainty** | **Importance** |
| --- | --- | --- | --- | --- | --- | --- | --- | --- | --- | --- | --- | --- |
| **№ of studies** | **Study design** | **Risk of bias** | **Inconsistency** | **Indirectness** | **Imprecision** | **Other considerations** | **Care bundle** | **control** | **Relative (95% CI)** | **Absolute (95% CI)** |  |  |
| 5 | observational studies | not serious | not serious | serious^a^ | not serious | none | 674/5378 (12.5%) | 1438/8209 (17.5%) | **OR 0.77** (0.69 to 0.86) | **35 fewer per 1,000** (from 47 fewer to 21 fewer) | ⨁◯◯◯ Very low |  |

**CI:** confidence interval; **OR:** odds ratio

#### Explanations

1. Population: hospitalized patients, ICU patients, cardiac surgery

**9.2.** Moderate-severe **AKI**

| **Certainty assessment** | | | | | | | **№ of patients** | | **Effect** | | **Certainty** | **Importance** |
| --- | --- | --- | --- | --- | --- | --- | --- | --- | --- | --- | --- | --- |
| **№ of studies** | **Study design** | **Risk of bias** | **Inconsistency** | **Indirectness** | **Imprecision** | **Other considerations** | **Care bundle** | **control** | **Relative (95% CI)** | **Absolute (95% CI)** |  |  |
| 11 | observational studies | not serious | not serious | serious^a^ | not serious | none | 446/6241 (7.1%) | 736/9005 (8.2%) | **OR 0.65** (0.51 to 0.82) | **27 fewer per 1,000** (from 38 fewer to 14 fewer) | ⨁◯◯◯ Very low |  |

**CI:** confidence interval; **OR:** odds ratio

#### Explanations

1. population: cardiac surgery, non-cardiac surgery, ICU patients, AKI patients, hospitalized patients, ICU patients

**9.3.** Moderate-severe **AKI (Biomarker-guided studies)**

| **Certainty assessment** | | | | | | | **№ of patients** | | **Effect** | | **Certainty** | **Importance** |
| --- | --- | --- | --- | --- | --- | --- | --- | --- | --- | --- | --- | --- |
| **№ of studies** | **Study design** | **Risk of bias** | **Inconsistency** | **Indirectness** | **Imprecision** | **Other considerations** | **Care bundle** | **control** | **Relative (95% CI)** | **Absolute (95% CI)** |  |  |
| 7 | observational studies | not serious | not serious | serious^a^ | not serious | none | 101/1128 (9.0%) | 155/1170 (13.2%) | **OR 0.57** (0.42 to 0.76) | **52 fewer per 1,000** (from 72 fewer to 28 fewer) | ⨁◯◯◯ Very low |  |

**CI:** confidence interval; **OR:** odds ratio

#### Explanations

1. Population: cardiac surgery, emergency department, intensive care unit, noncardiac surgery

**9.4.** Moderate-severe **AKI (studies without biomarker use)**

| **Certainty assessment** | | | | | | | **№ of patients** | | **Effect** | | **Certainty** | **Importance** |
| --- | --- | --- | --- | --- | --- | --- | --- | --- | --- | --- | --- | --- |
| **№ of studies** | **Study design** | **Risk of bias** | **Inconsistency** | **Indirectness** | **Imprecision** | **Other considerations** | **Care bundle** | **control** | **Relative (95% CI)** | **Absolute (95% CI)** |  |  |
| 4 | observational studies | not serious | serious^a^ | serious | not serious | none | 345/5113 (6.7%) | 581/7835 (7.4%) | **OR 0.71** (0.51 to 0.99) | **20 fewer per 1,000** (from 35 fewer to 1 fewer) | ⨁◯◯◯ Very low |  |

**CI:** confidence interval; **OR:** odds ratio

#### Explanations

1. I^2^: 59%
   1. **Renal Replacement Therapy (RRT)**

| **Certainty assessment** | | | | | | | **№ of patients** | | **Effect** | | **Certainty** | **Importance** |
| --- | --- | --- | --- | --- | --- | --- | --- | --- | --- | --- | --- | --- |
| **№ of studies** | **Study design** | **Risk of bias** | **Inconsistency** | **Indirectness** | **Imprecision** | **Other considerations** | **Care bundle** | **control** | **Relative (95% CI)** | **Absolute (95% CI)** |  |  |
| 8 | observational studies | not serious | not serious | serious | not serious | none | 67/2873 (2.3%) | 135/3710 (3.6%) | **OR 0.63** (0.46 to 0.88) | **13 fewer per 1,000** (from 19 fewer to 4 fewer) | ⨁◯◯◯ Very low |  |

**CI:** confidence interval; **OR:** odds ratio

**9.6.RRT (Biomarker-guided studies)**

| **Certainty assessment** | | | | | | | **№ of patients** | | **Effect** | | **Certainty** | **Importance** |
| --- | --- | --- | --- | --- | --- | --- | --- | --- | --- | --- | --- | --- |
| **№ of studies** | **Study design** | **Risk of bias** | **Inconsistency** | **Indirectness** | **Imprecision** | **Other considerations** | **Care bundle** | **control** | **Relative (95% CI)** | **Absolute (95% CI)** |  |  |
| 6 | observational studies | not serious | not serious | serious | not serious | none | 22/585 (3.8%) | 33/594 (5.6%) | **OR 0.70** (0.40 to 1.25) | **15 fewer per 1,000** (from 33 fewer to 13 more) | ⨁◯◯◯ Very low |  |

**CI:** confidence interval; **OR:** odds ratio

**9.7.RRT (studies without biomarker use)**

| **Certainty assessment** | | | | | | | **№ of patients** | | **Effect** | | **Certainty** | **Importance** |
| --- | --- | --- | --- | --- | --- | --- | --- | --- | --- | --- | --- | --- |
| **№ of studies** | **Study design** | **Risk of bias** | **Inconsistency** | **Indirectness** | **Imprecision** | **Other considerations** | **Care bundle** | **control** | **Relative (95% CI)** | **Absolute (95% CI)** |  |  |
| 2 | observational studies | not serious | serious^a^ | serious | not serious | none | 45/2286 (2.0%) | 102/3118 (3.3%) | **OR 0.60** (0.35 to 1.04) | **13 fewer per 1,000** (from 21 fewer to 1 more) | ⨁◯◯◯ Very low |  |

**CI:** confidence interval; **OR:** odds ratio; RRT = renal replacement therapy

#### Explanations

1. I^2^: 59%

**9.8.Mortality**

| **Certainty assessment** | | | | | | | **№ of patients** | | **Effect** | | **Certainty** | **Importance** |
| --- | --- | --- | --- | --- | --- | --- | --- | --- | --- | --- | --- | --- |
| **№ of studies** | **Study design** | **Risk of bias** | **Inconsistency** | **Indirectness** | **Imprecision** | **Other considerations** | **Care bundle** | **control** | **Relative (95% CI)** | **Absolute (95% CI)** |  |  |
| 11 | observational studies | not serious | not serious | serious^a^ | not serious | none | 313/5438 (5.8%) | 793/7413 (10.7%) | **OR 0.88** (0.75 to 1.02) | **12 fewer per 1,000** (from 25 fewer to 2 more) | ⨁◯◯◯ Very low |  |

**CI:** confidence interval; **OR:** odds ratio

#### Explanations

1. population: cardiac surgery, non-cardiac surgery, ICU patients, AKI patients, hospitalized patients, ICU patients

**9.9.Mortality (Biomarker-guided studies)**

| **Certainty assessment** | | | | | | | **№ of patients** | | **Effect** | | **Certainty** | **Importance** |
| --- | --- | --- | --- | --- | --- | --- | --- | --- | --- | --- | --- | --- |
| **№ of studies** | **Study design** | **Risk of bias** | **Inconsistency** | **Indirectness** | **Imprecision** | **Other considerations** | **Care bundle** | **control** | **Relative (95% CI)** | **Absolute (95% CI)** |  |  |
| 7 | observational studies | not serious | not serious | serious^a^ | not serious | none | 29/999 (2.9%) | 38/1027 (3.7%) | **OR 0.76** (0.46 to 1.25) | **9 fewer per 1,000** (from 20 fewer to 9 more) | ⨁◯◯◯ Very low |  |

**CI:** confidence interval; **OR:** odds ratio

#### Explanations

1. Population: cardiac surgery, emergency department, intensive care unit, noncardiac surgery

**9.10. Mortality (Studies without biomarker use)**

| **Certainty assessment** | | | | | | | **№ of patients** | | **Effect** | | **Certainty** | **Importance** |
| --- | --- | --- | --- | --- | --- | --- | --- | --- | --- | --- | --- | --- |
| **№ of studies** | **Study design** | **Risk of bias** | **Inconsistency** | **Indirectness** | **Imprecision** | **Other considerations** | **Care bundle** | **control** | **Relative (95% CI)** | **Absolute (95% CI)** |  |  |
| 4 | observational studies | not serious | serious^a^ | serious^b^ | not serious | none | 284/4439 (6.4%) | 755/6386 (11.8%) | **OR 0.90** (0.71 to 1.14) | **11 fewer per 1,000** (from 31 fewer to 14 more) | ⨁◯◯◯ Very low |  |

**CI:** confidence interval; **OR:** odds ratio

#### Explanations

a. I^2^: 52%

b. Population: hospitalized patients, ICU patients

1. **Supplementary Figure 4a-d. Subgroup analyses on cardiovascular surgery**

A subgroup analysis focusing on patients who underwent cardiovascular surgery

**Supplementary Figure 4a. Forest plot of RRT from application of care bundles vs. usual care with focus on subgroup analysis of patients post cardiovascular surgery**


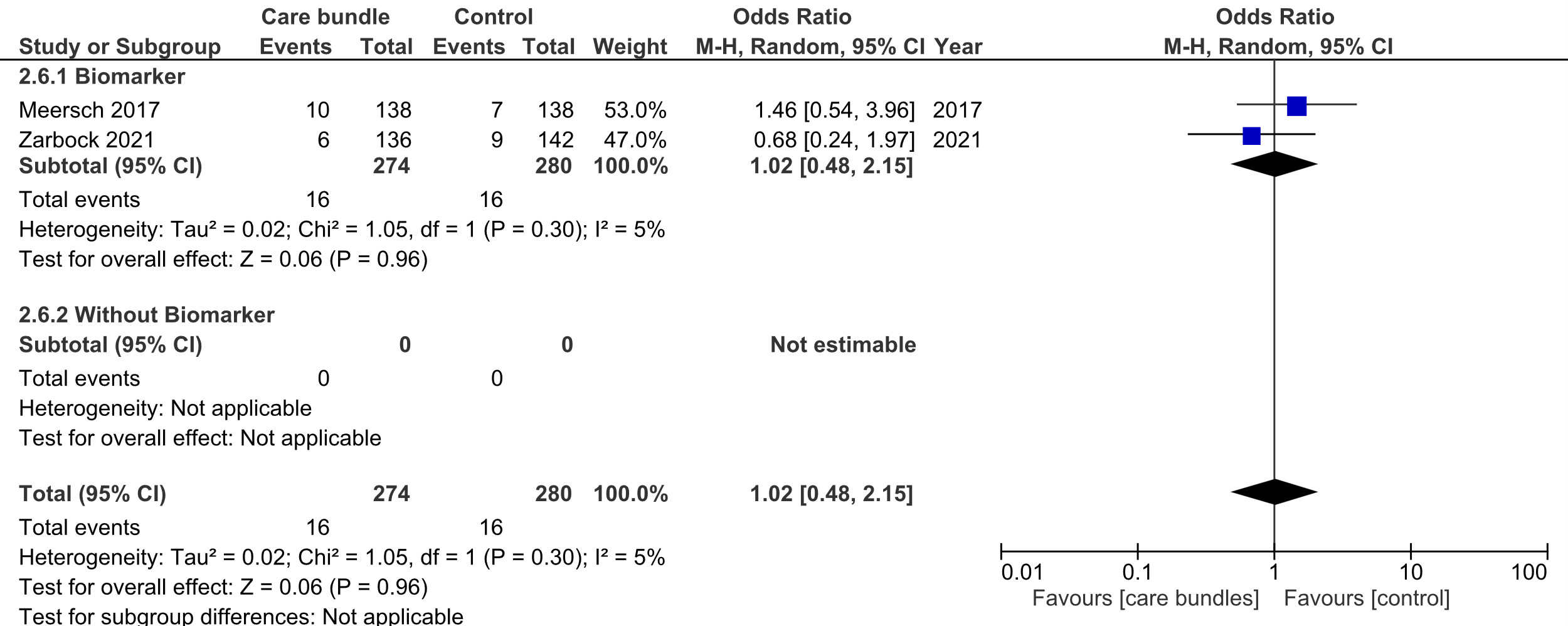


**Supplementary Figure 4b. Forest plot of mortality from application of care bundles vs. usual care with focus on subgroup analysis of patients post cardiovascular surgery**


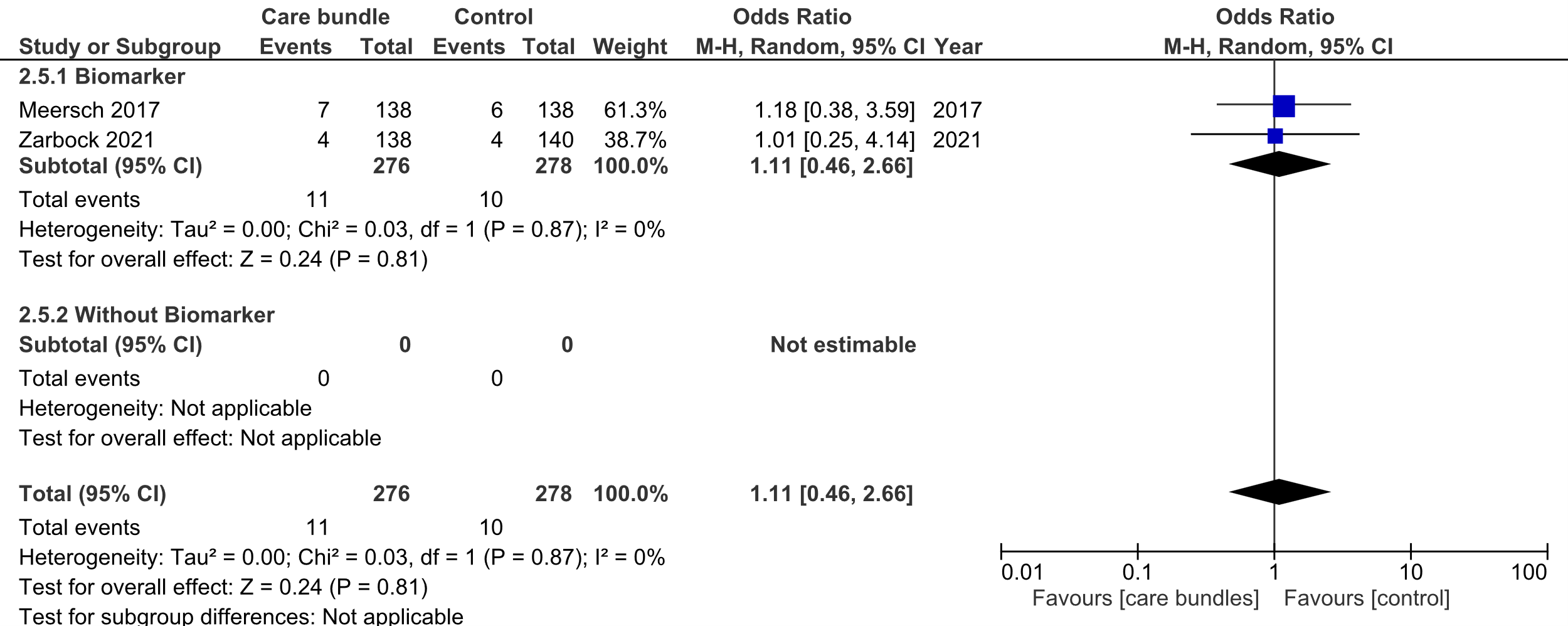


**Supplementary Figure 4c. Forest plot of moderate-severe AKI from care bundle vs. usual care focused subgroup analysis on cardiovascular surgery**


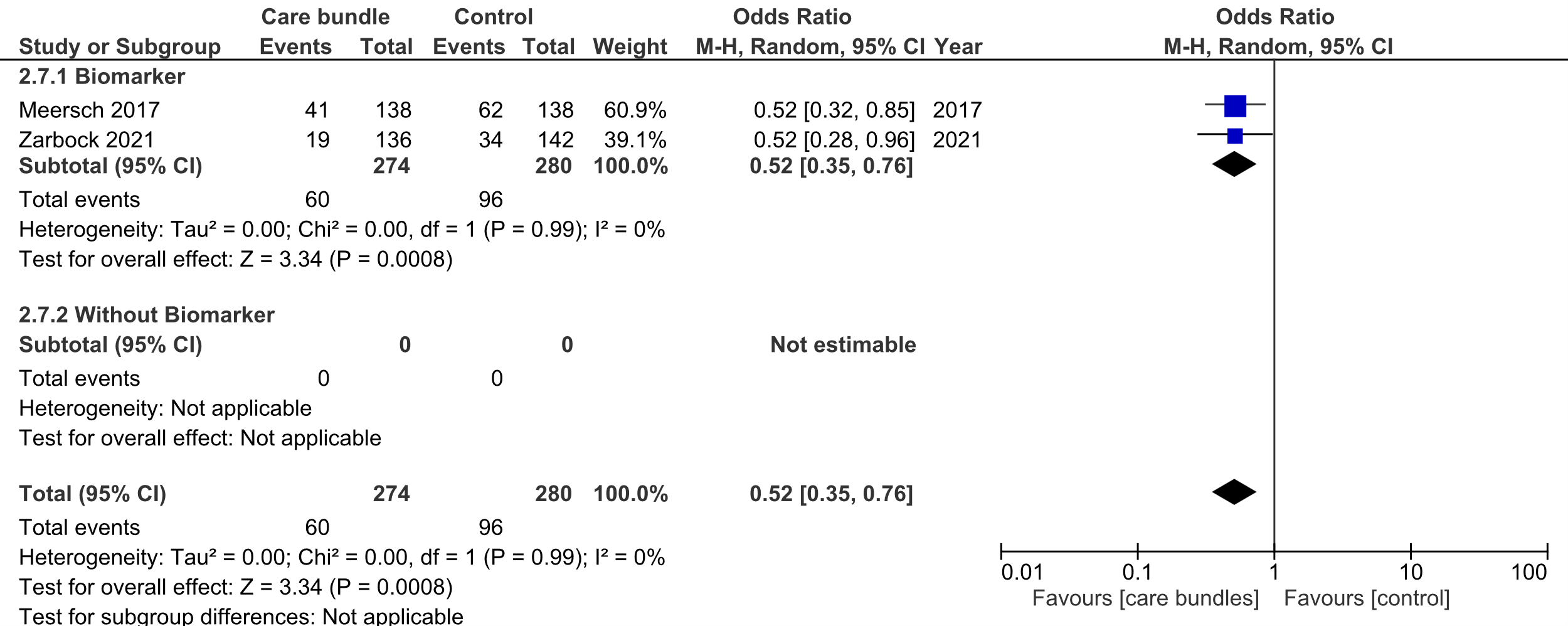


**Supplementary Figure 4d. Forest plot of MAKE from care bundle vs. usual care focused subgroup analysis on cardiovascular surgery**


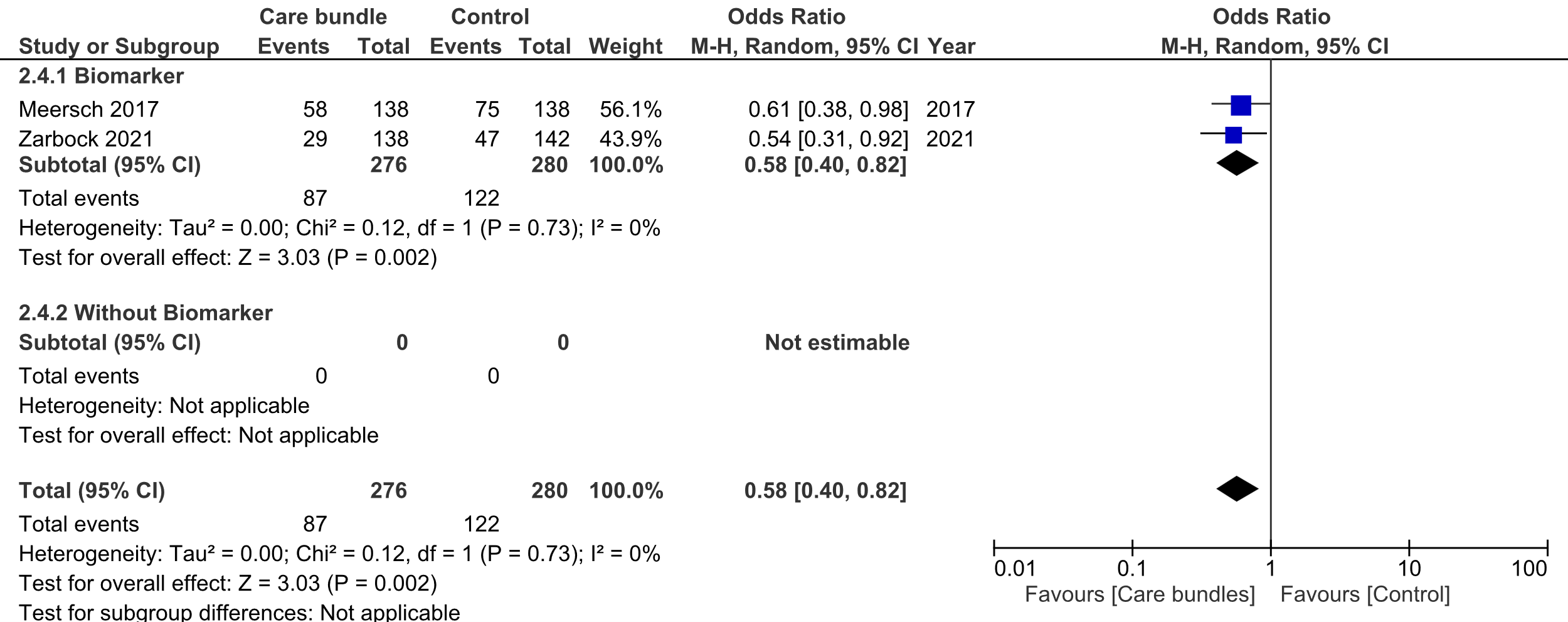


1. **Supplementary Figure 5.** A network meta-analysis was conducted to compare the effectiveness of MAKE occurrence between patients who received non-biomarker-guided bundle care implementation, biomarker-guided bundle care implementation, and usual care without bundle. Results showed that biomarker-guided bundle care implementation was more likely to be ranked higher in terms of its effectiveness.


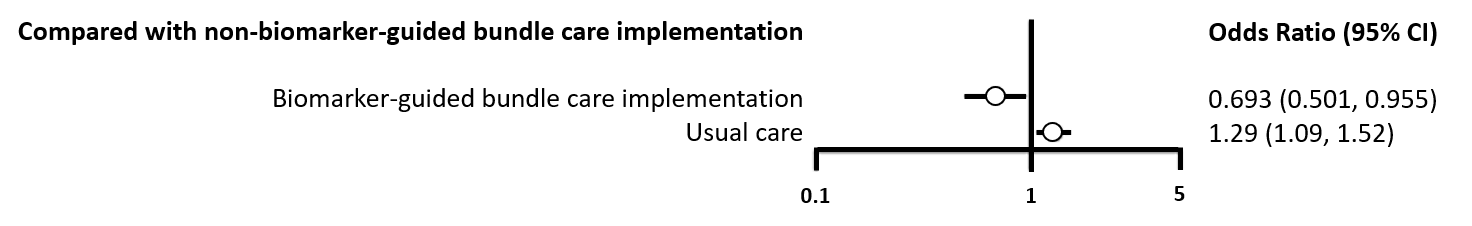


1. **Supplementary Figure 6a-d. Hierarchical summary receiver operating characteristic (HSROC) curves**

The surface under the cumulative ranking (SUCRA) curve representing the relative effectiveness of non-biomarker-guided bundle care implementation, biomarker-guided bundle care implementation, and usual care without bundle. It is presented as a plot, with the SUCRA score as the *y*-axis and the cumulative probability of the intervention rank as the *x*-axis. A higher SUCRA score indicates a better outcome, meaning that a treatment group is more likely to be ranked higher in terms of its effectiveness. (A) SUCRA plot, (B) Cumulative probability of the intervention rank and SUCRA.

**Supplementary Figure 6a.**  SUCRA plot.


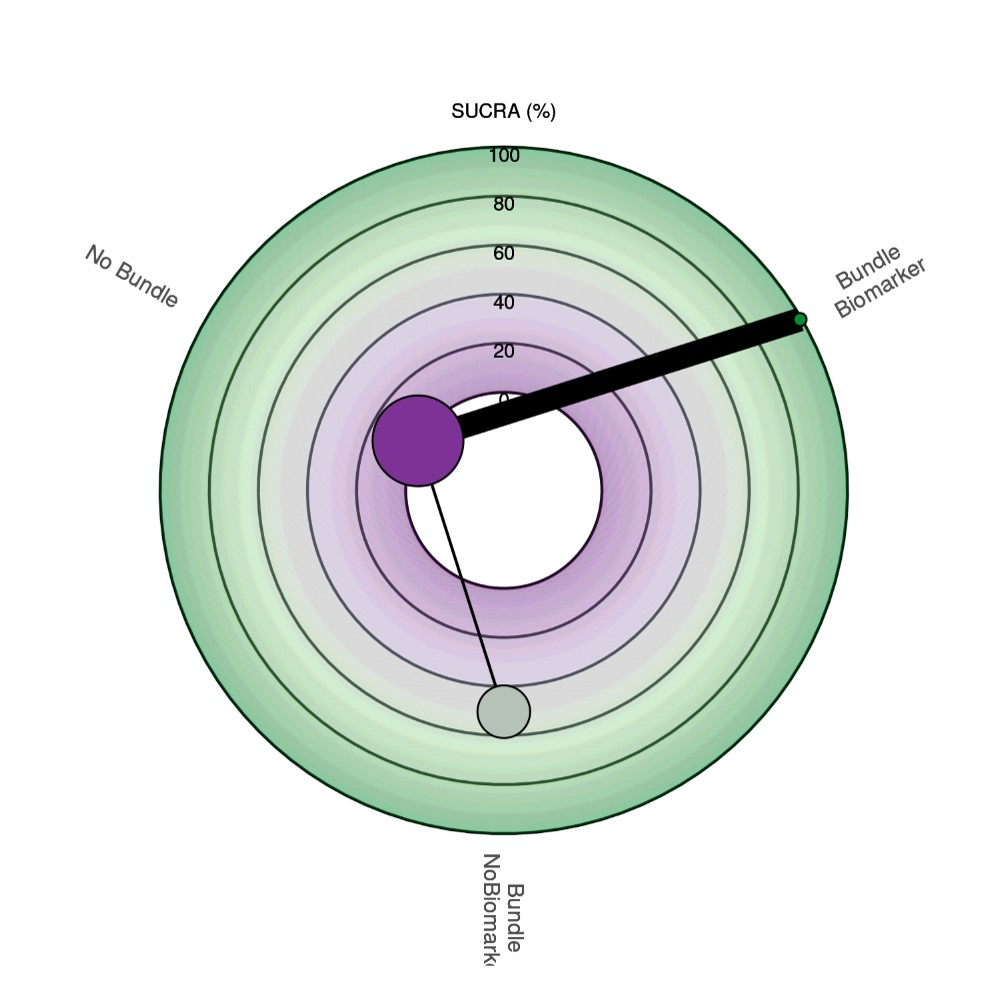


**Supplementary Figure 6b.** Cumulative probability of the intervention rank and SUCRA.


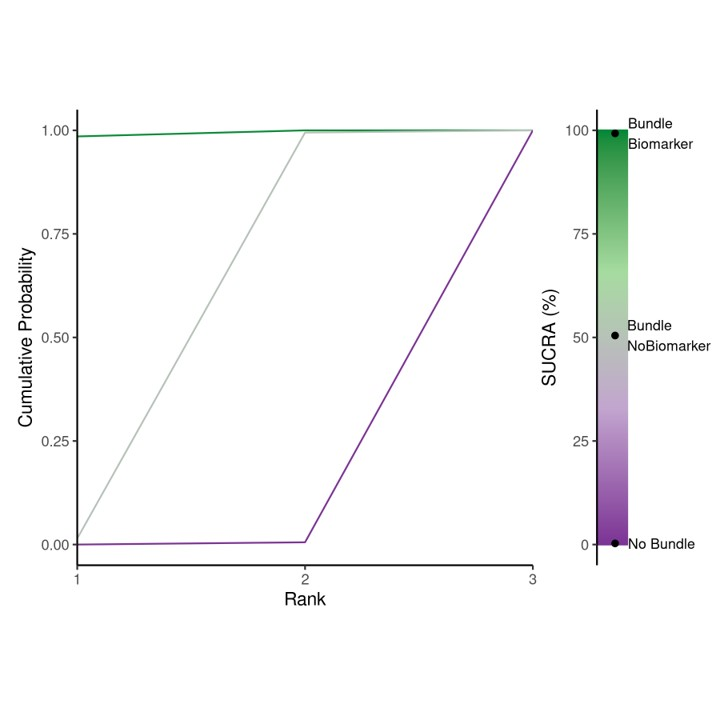


**References:**

1. Kolhe NV, Staples D, Reilly T, Merrison D, McIntyre CW, Fluck RJ, et al. Impact of Compliance with a Care Bundle on Acute Kidney Injury Outcomes: A Prospective Observational Study. PLoS One. 2015;10(7):e0132279.

2. Kolhe NV, Reilly T, Leung J, Fluck RJ, Swinscoe KE, Selby NM, et al. A simple care bundle for use in acute kidney injury: a propensity score-matched cohort study. Nephrol Dial Transplant. 2016;31(11):1846-54.

3. Meersch M, Schmidt C, Hoffmeier A, Van Aken H, Wempe C, Gerss J, et al. Prevention of cardiac surgery-associated AKI by implementing the KDIGO guidelines in high risk patients identified by biomarkers: the PrevAKI randomized controlled trial. Intensive Care Med. 2017;43(11):1551-61.

4. Göcze I, Jauch D, Götz M, Kennedy P, Jung B, Zeman F, et al. Biomarker-guided Intervention to Prevent Acute Kidney Injury After Major Surgery: The Prospective Randomized BigpAK Study. Ann Surg. 2018;267(6):1013-20.

5. Kapoor PM, Karanjkar A, Magoon R, Taneja S, Das S, Malik V, et al. Effect of goal-directed therapy on post-operative neutrophil gelatinase-associated lipocalin profile in patients undergoing on-pump coronary artery surgery. Indian J Thorac Cardiovasc Surg. 2019;35(3):445-52.

6. Schanz M, Wasser C, Allgaeuer S, Schricker S, Dippon J, Alscher MD, et al. Urinary [TIMP-2]·[IGFBP7]-guided randomized controlled intervention trial to prevent acute kidney injury in the emergency department. Nephrol Dial Transplant. 2019;34(11):1902-9.

7. Engelman DT, Crisafi C, Germain M, Greco B, Nathanson BH, Engelman RM, et al. Using urinary biomarkers to reduce acute kidney injury following cardiac surgery. J Thorac Cardiovasc Surg. 2020;160(5):1235-46.e2.

8. Koeze J, van der Horst ICC, Wiersema R, Keus F, Dieperink W, Cox EGM, et al. Bundled care in acute kidney injury in critically ill patients, a before-after educational intervention study.

9. Zarbock A, Küllmar M, Ostermann M, Lucchese G, Baig K, Cennamo A, et al. Prevention of Cardiac Surgery-Associated Acute Kidney Injury by Implementing the KDIGO Guidelines in High-Risk Patients Identified by Biomarkers: The PrevAKI-Multicenter Randomized Controlled Trial. Anesth Analg. 2021;133(2):292-302.

10. Halmy L, Riedel J, Zeman F, Tege B, Linder V, Gnewuch C, et al. Renal Recovery after the Implementation of an Electronic Alert and Biomarker-Guided Kidney-Protection Strategy following Major Surgery. J Clin Med. 2021;10(21).

11. Couturier C, Maillard N, Mariat C, Morel J, Palao JC, Bouchet JB, et al. Prevention of cardiac surgery-associated acute kidney injury by risk stratification using (TIMP-2)*(IGFBP7). Biomark Med. 2021;15(14):1201-10.

12. Bourdeaux C, Ghosh E, Atallah L, Palanisamy K, Patel P, Thomas M, et al. Impact of a computerized decision support tool deployed in two intensive care units on acute kidney injury progression and guideline compliance: a prospective observational study.

13. Kotwal S, Herath S, Erlich J, Boardman S, Qian J, Lawton P, et al. Electronic alerts and a care bundle for acute kidney injury - an Australian cohort study. Nephrol Dial Transplant. 2022.
